# Dysregulation of bile acids increases the risk for preterm birth in pregnant women

**DOI:** 10.1038/s41467-020-15923-4

**Published:** 2020-04-30

**Authors:** Sangmin You, Ai-Min Cui, Syed F. Hashmi, Xinmu Zhang, Christina Nadolny, Yuan Chen, Qiwen Chen, Xin Bush, Zachary Hurd, Winifer Ali, Gang Qin, Ruitang Deng

**Affiliations:** 1 Department of Biomedical and Pharmaceutical Sciences, College of Pharmacy, University of Rhode Island, 7 Greenhouse Road, Kingston, RI 02881 USA; 2 Nantong Maternal and Child Health Hospital, Nantong University, Nantong, China; 3 Department of Epidemiology and Biostatistics, School of Public Health, Nantong University, 226006 Nantong, Jiangsu Province China

**Keywords:** Reproductive disorders, Liver diseases, Non-alcoholic fatty liver disease

## Abstract

Preterm birth (PTB) is the leading cause of perinatal mortality and newborn complications. Bile acids are recognized as signaling molecules regulating a myriad of cellular and metabolic activities but have not been etiologically linked to PTB. In this study, a hospital-based cohort study with 36,755 pregnant women is conducted. We find that serum total bile acid levels directly correlate with the PTB rates regardless of the characteristics of the subjects and etiologies of liver disorders. Consistent with the findings from pregnant women, PTB is successfully reproduced in mice with liver injuries and dysregulated bile acids. More importantly, bile acids dose-dependently induce PTB with minimal hepatotoxicity. Furthermore, restoring bile acid homeostasis by farnesoid X receptor activation markedly reduces PTB and dramatically improves newborn survival rates. The findings thus establish an etiologic link between bile acids and PTB, and open an avenue for developing etiology-based therapies to prevent or delay PTB.

## Abstract (web summary)

Preterm birth (PTB) is the leading cause of perinatal mortality and newborn complications with limited treatment options. Here the authors show that dysregulation of bile acids increases risk for PTB in pregnant women while restoring bile acid homeostasis delays or prevents PTB in the mouse models.

## Introduction

Preterm birth (PTB) is defined as babies born before 37 weeks of pregnancy (gestation) and is the leading cause of perinatal mortality worldwide^1,2^. Surviving preterm infants are also at risk for neurological, respiratory, and gastrointestinal complications; metabolic syndrome; and cardiovascular disease^3–5^. The prevalence of PTB ranges from 5% to 18% globally^6^. In the United States, one out of ten newborns is delivered preterm and the PTB rates exhibit an uptrend with unknown reasons^7^.

The underlying mechanisms by which PTB is induced are complex and multi-factorial^8,9^. Despite current research efforts, our understanding on the underlying mechanisms for PTB remains limited. As a consequence, current available interventions for preventing PTB show inconsistent benefits^10,11^. Bile acids are synthesized in the liver and their homeostasis is maintained through a tightly regulated enterohepatic circulation mainly through the complex farnesoid X receptor (FXR) signaling pathways^12,13^. Bile acids are recently recognized as signaling molecules to regulate a variety of cellular and metabolic activities^14–16^. In the studies of pregnant women with intrahepatic cholestasis of pregnancy (ICP), it is recognized that elevated bile acids are associated with increased risk for PTB^17–19^. However, a direct etiological link between bile acids and PTB has not been established and it remains to be determined whether liver injury itself or elevated bile acids as a result of liver injury induce PTB.

In this study, a hospital-based cohort study with pregnant women is conducted. PTB rates and serum total bile acids (sTBAs) are determined and their correlations are evaluated. Using mouse models, PTB is successfully reproduced with liver injuries or bile acid challenge alone. Furthermore, restoring bile acid homeostasis by FXR activation markedly reduces PTB and dramatically improves newborn survival. The findings establish a direct etiological link between bile acids and PTB and open an avenue for developing mechanism-based therapies to prevent or delay PTB.

## Results

### Participant characteristics

Among the 36,755 pregnant women enrolled in the study, 36,612 subjects were ethnic Han Chinese women (99.61%), while 143 subjects were other ethnic minority women (0.39%). Twenty-two thousand five hundred and seventy-one women (61.41%) were primipara, while 14,184 women (38.59%) were multipara. Among the multipara, 652 women had a history of PTB, while 23 women had a history of stillbirth. The patients diagnosed with ICP received ursodeoxycholic acid at a dose of 15 mg kg^−1^ day^−1^ until delivery. The cervical length of the subjects with PTB was not evaluated. The average gestation age when PTB occurred was 34 ± 2.3 weeks. Most of the PTBs occurred at the gestation ages between 33 and 36 weeks (*n* = 2496, 86.8%) with 55 subjects (1.9%) having PTB at gestation ages <28 weeks and 324 women (11.3%) having PTB at gestation ages 28–32 weeks.

### sTBA levels positively correlated with the rates of PTB

**Fig. 1:**
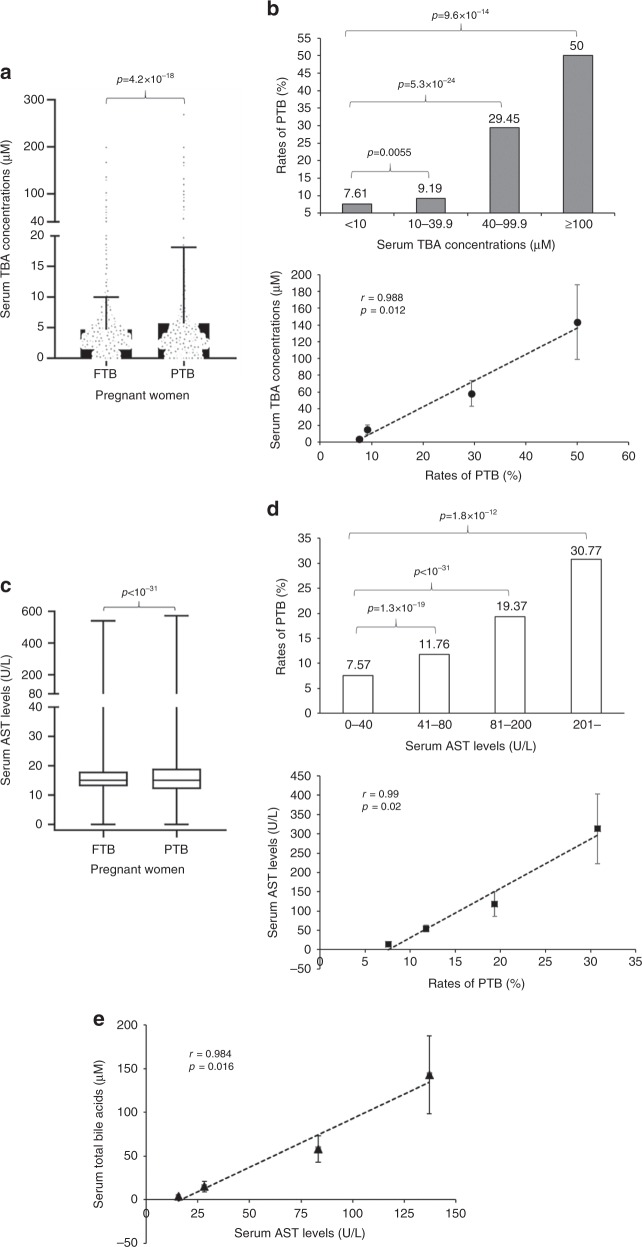
Elevated sTBAs in pregnant women with PTB. **a** sTBA levels in pregnant women with FTB (*n* = 33,880) and PTB (*n* = 2875). Bar chart shows the mean ± SD with small dots representing the data point distribution. **b** As sTBA levels elevated from <10 (*n* = 34,227) to 10–39.9 (*n* = 2362), 40–99.9 (*n* = 146) and ≥100 μM (*n* = 20), the PTB rates gradually increased. The PTB rates directly correlated with the sTBA concentrations. Data points in the lower panel are presented as mean ± SD. **c** Serum AST levels in pregnant women with FTB (*n* = 33,880) and PTB (*n* = 2875). The box and whisker plots show the maximum, minimum data points, median value, and 75th and 25th quartile. **d** Various AST levels of 0–40 (*n* = 35,360), 41–80 (*n* = 1046), 81–200 (*n* = 284), and >200 U L^−1^ (*n* = 65) with corresponding PTB rates. The PTB rates directly correlated with AST levels. Data points in the lower panel are presented as mean ± SD. **e** sTBA concentrations of <10 (*n* = 34,227), 10–39.9 (*n* = 2362), 40–99.9 (*n* = 146), and ≥100 μM (*n* = 20) directly correlated with the AST levels. Data points are presented as mean ± SD. Student’s *t* test for pairwise comparison (two-sided) and one-way ANOVA, followed by Tukey post hoc test were applied for statistical analysis. Pearson correlation analysis was applied to determine the correlation coefficient and associated *p* values. Source data are provided as a Source Data file.

Among 36,755 pregnant women, 2875 subjects experienced PTB while 33,880 subjects had full-term birth (FTB). The sTBAs were significantly increased from 4.68 ± 5.31 μM in FTB to 5.69 ± 12.45 μM in PTB subjects (*p* = 4.2 × 10^−18^) (Fig. 1a). When sTBAs were elevated from <10 (*n* = 34,227) to 10–39.9 (*n* = 2362), 40–99.9 (*n* = 146), and ≥100 μM (*n* = 20), the rates of PTB were significantly increased from 7.6% to 9.2% (relative risk (RR) 1.21, 95% confidence interval (CI) 1.05–1.39) (*p* = 0.0055), 29.5% (RR 3.87, 95% CI 2.86–5.23) (*p* = 5.3 × 10^−24^), and 50% (RR 6.57, 95% CI 3.53–12.22) (*p* = 9.6 × 10^−14^), respectively (Fig. 1b and Supplementary Table 1). Correlation analysis confirmed that sTBA concentrations directly correlated with the PTB rates (*r* = 0.988; *p* = 0.012; Fig. 1b).

PTB is clinically categorized into spontaneous (sPTB) and iatrogenic (medically indicated) PTB (iPTB). Among the 2875 PTB subjects, 1302 had sPTB (45.3%) while 1573 had iPTB (54.7%). Compared with FTB subjects, the sTBAs were significantly elevated in subjects with sPTB (*p* = 0.0027) and iPTB subjects (*p* = 2.6 × 10^−22^; Supplementary Fig. 1a). The sPTB rates were significantly raised from 3.5% in subjects with <10 μM sTBAs to 10.3% in subjects with sTBAs 40–99.9 μM (RR 2.9, 95% CI 1.74–4.83; *p* = 0.0001) and to 20% in subjects with sTBAs ≥100 μM (RR 5.65, 95% CI 2.12–15.07; *p* = 0.005) (Supplementary Fig. 1b and Supplementary Table 1). Consistently, sTBA levels positively correlated with the sPTB rates (*r* = 0.988, *p* = 0.007; Supplementary Fig. 1b). Similar to sPTB, the iPTB rates significantly increased from 4.2% to 6.2% (RR 1.51, 95% CI 1.28–1.80; *p* = 7.6 × 10^−8^), 19.2% (RR 4.71, 95% CI 3.24–6.85; *p* = 5.7 × 10^−21^), and 30% (RR 7.37, 95% CI 3.31–16.44; *p* = 4.7 × 10^−10^), respectively, when sTBA concentrations were elevated from <10 to 10–39.9, 40–99.9, and ≥100 μM (Supplementary Fig. 1c and Supplementary Table 1). sTBA levels were also directly correlated with the iPTB rates (*r* = 0.978, *p* = 0.002; Supplementary Fig. 1c).

Consistent with sTBA, aspartate aminotransferase (AST) levels, which serve as the markers for liver injuries, were significantly increased from 16.36 ± 14.70 U L^−1^ in FTB subjects to 20.35 ± 28.56 U L^−1^ in PTB subjects (*p* < 10^−31^; Fig. 1c). When AST levels increased from 0–40 (*n* = 35,360) to 41–80 (*n* = 1046), 81–200 (*n* = 284), and >200 U L^−1^ (*n* = 65), the PTB rates were significantly increased from 7.57% to 11.76% (*p* = 1.3 × 10^−19^), 19.37% (*p* < 10^−31^), and 30.77% (*p* = 1.8 × 10^−12^), respectively (Fig. 1d). AST levels directly correlated with the PTB rates (*r* = 0.985, *p* = 0.015; Fig. 1d) and sTBA concentrations (Fig. 1e). Consistently, serum AST levels were significantly increased in subjects with sPTB (*p* = 1.7 × 10^−5^) and iPTB (*p* < 10^−31^) and directly correlated with the rates of sPTB (*r* = 0.968, *p* = 0.032) and iPTB (*r* = 0.964, *p* = 0.036) (Supplementary Fig. 1d–f). In addition, a positive correlation was detected between serum AST and sTBA levels in subjects with sPTB (*r* = 0.98, *p* = 0.02; Supplementary Fig. 1g). However, no significant correlation was detected between serum AST and TBA levels in subjects with iPTB (*r* = 0.909, *p* = 0.091; Supplementary Fig. 1h).

Consistent with findings for AST, the PTB rates were significantly increased when other liver injury markers were elevated. As alanine aminotransferase (ALT) levels were elevated from 0–40 to 41–80, 81–200, and >200 U L^−1^, the PTB rates were increased from 8.8% to 9.6% (*p* = 0.25), 14.0% (*p* = 5 × 10^−5^), and 27.7% (*p* = 2.2 × 10^−17^), respectively (Supplementary Fig. 1i). When total bilirubin (TBiL) concentrations were elevated from <17.1 to 17.2–34.2 μM L^−1^, the PTB rates remains unchanged. However, when TBiL concentrations were further elevated to >34.3 μM L^−1^, the PTB rates were markedly increased from 8.7% to 37.5% (*p* = 1.3 × 10^−12^; Supplementary Fig. 1j). Similarly, as serum gamma-glutamyl transpeptidase (GGT) levels elevated from <50 to 51–100 and >100 U L^−1^, the PTB rates were significantly increased from 8.8% to 23.3% (*p* = 2.2 × 10^−20^) and 23.7% (*p* = 3.4 × 10^−9^), respectively (Supplementary Fig. 1k).

### Characteristics of pregnant women, sTBA levels, and PTB rates

Among the characteristics of the subjects, maternal age^20–23^, and pre-pregnancy body mass index (BMI)^24–27^ had significant impacts on PTB. We performed correlation analyses between sTBA concentrations and PTB rates in subjects with different maternal ages or pre-pregnancy BMIs.

**Fig. 2:**
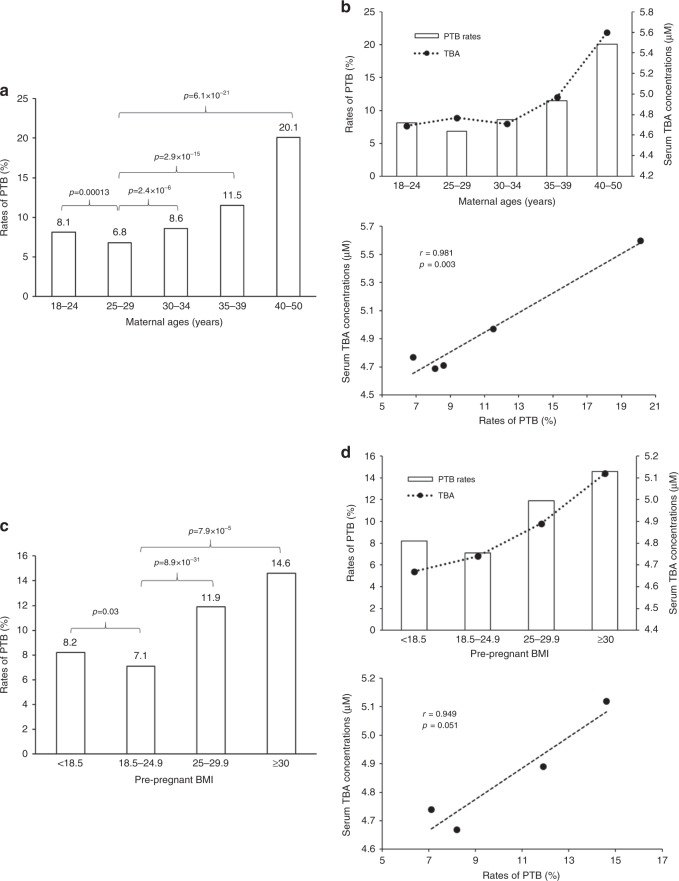
Advanced maternal age and high pre-pregnancy BMI increased the risk for PTB with elevated sTBA. **a** The PTB rates of five groups of pregnant women at age 18–24 (*n* = 9,000), 25–29 (*n* = 19,289), 30–34 (*n* = 6122), 35–39 (*n* = 2025), and 40–50 (*n* = 319) years. **b** Serum TBA exhibited an uptrend as maternal age advanced and directly correlated with the PTB rates among the five age groups with the same sample sizes described in **a**. **c** The PTB rates of four groups of pregnant women with various BMIs, <18.5 (*n* = 2973), 18.5–24.9 (*n* = 29,004), 25–29.9 (*n* = 4593). and ≥30 (*n* = 185). **d** Serum TBA concentrations were elevated in overweight/obese subjects and positively correlated with the PTB rates among the four BMI groups with the same sample sizes described in **c**. One-way ANOVA, followed by Tukey post hoc test, and Pearson correlation test were applied for statistical analysis. Source data are provided as a Source Data file.

The subjects were divided into five age groups, 18–24, 25–29, 30–34, 35–39, and 40–50 years. Subjects at ages 25–29 years had the largest population (*n* = 19,289) and the lowest PTB rate (6.8%) (Fig. 2a and Supplementary Table 2). As the maternal age advanced, the PTB rates increased from 6.8% to 8.6% (*p* = 2.4 × 10^−6^), 11.5% (*p* = 2.9 × 10^−15^), and 20.1% (*p* = 6.1 × 10^−21^) in the age groups 30–34 (*n* = 6122), 35–39 (*n* = 2025), and 40–50 (*n* = 319), respectively. The youngest age group (age 18–24, *n* = 9000) also had a significantly increased PTB rate (8.1%) (*p* = 1.3 × 10^−4^). sTBAs showed an uptrend as maternal age advanced and directly correlated with the PTB rates among the five age groups (*r* = 0.981, *p* = 0.003; Fig. 2b).

Based on the BMIs, the subjects were categorized into four groups. Among the 36,755 pregnant women, 78.91% subjects had healthy body weight (BMI 18.5–24.9, *n* = 29,004) with the lowest PTB rate of 7.1% (Fig. 2c). When pregnant women become overweight (BMI 25–29.9, *n* = 4593) and obese (BMI ≥ 30, *n* = 185), the PTB rates were significantly increased to 11.9% (*p* = 8.9 × 10^−31^) and 14.6% (*p* = 7.9 × 10^−5^), respectively. On the other hand, underweight subjects (BMI < 18.5, *n* = 2973) also had a significantly increased PTB rate of 8.2% (*p* = 0.03). More importantly, sTBAs exhibited an increased trend as BMI increased and directly correlated with the PTB rates among the four BMI groups (*r* = 0.949; *p* = 0.05).

Similar to sTBA, AST levels gradually increased as maternal age advanced or BMI increased and positively correlated with the PTB rates (*r* = 0.988, *p* = 0.002 or *r* = 0.966, *p* = 0.034, respectively; Supplementary Fig. 2a, b). As expected, the AST and sTBA levels were directly correlated among the five age groups (*r* = 0.983, *p* = 0.003) and four BMI groups (*r* = 0.976, *p* = 0.024) (Supplementary Fig. 2c, d).

### Liver disorders of pregnant women, sTBA levels, and PTB rates

**Fig. 3:**
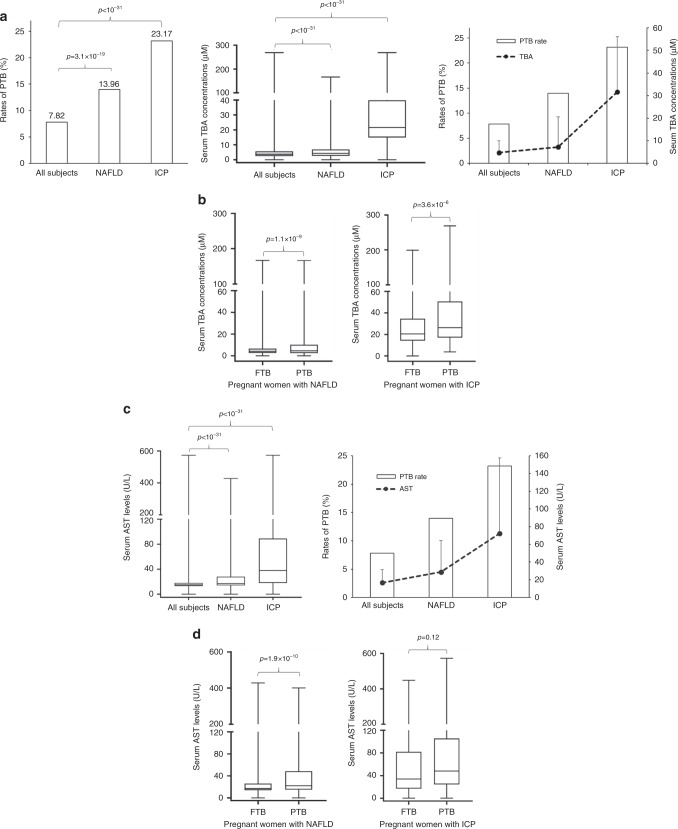
Pregnant women with NAFLD and ICP had increased risk for PTB with elevated sTBAs. **a** The PTB rates, sTBA levels, and their correlations in all subjects (*n* = 36,755) and the subjects with NAFLD (*n* = 1554) or ICP (*n* = 505). The box and whisker plots of the middle panel show the maximum, minimum data points, median value, and 75th and 25th quartile. Data points for serum TBA are presented as mean ± SD in the right panel. **b** The sTBA levels in the subjects with FTB (*n* = 1337 for NAFLD, *n* = 388 for ICP) and PTB (*n* = 217 for NAFLD, *n* = 117 for ICP) among the pregnant women with NAFLD or ICP. The box and whisker plots show the maximum, minimum data points, median value, and 75th and 25th quartile. **c** Serum AST levels in all subjects (*n* = 36,755) and subjects with NAFLD (*n* = 1554) or ICP (*n* = 505). The AST levels correlated with the rates of PTB. The box and whisker plots in the left panel show the maximum, minimum data points, median value, and 75th and 25th quartile. The data points for AST levels are presented as mean ± SD in the right panel. **d** The AST levels in subjects with FTB (*n* = 1337 for NAFLD and *n* = 388 for ICP) and PTB (*n* = 217 for NAFLD and *n* = 117 for ICP) among the pregnant women with NAFLD or ICP. The box and whisker plots show the maximum, minimum data points, median value, and 75th and 25th quartile. Student’s *t* test for pairwise comparison or one-way ANOVA, followed by Tukey post hoc test for multiple group comparison were applied for statistical analysis. Source data are provided as a Source Data file.

Among the 36,755 subjects, 1805 were diagnosed having non-alcoholic fatty liver disease (NAFLD), a rate of 4.91%, while 505 were diagnosed having ICP, a rate of 1.37%. Compared with the rate of 7.82% PTB in all the subjects, pregnant women with NAFLD and ICP had significantly increased PTB rates, 13.96% (*p* = 3.1 × 10^−19^) and 23.17% (*p* < 10^−31^), respectively (Fig. 3a). Such increases in PTB rates correlated with elevated sTBA concentrations in those subjects. The sTBA levels were significantly elevated from 4.8 ± 5.3 μM in all subjects to 7.3 ± 13.3 μM in NAFLD (*p* < 10^−31^) and 31.7 ± 24.4 μM in ICP subjects (*p* < 10^−31^) (Fig. 3a). Among the subjects with NAFLD, the sTBA levels were significantly higher in subjects with PTB (11.83 ± 22.01 μM, *n* = 217) than those with FTB (6.53 ± 10.28 μM, *n* = 1337) (*p* = 1.1 × 10^−9^; Fig. 3b). Similarly, among the pregnant women with ICP, significantly higher sTBA levels were detected in PTB subjects (42.92 ± 42.44 μM, *n* = 117) when compared to FTB subjects (28.33 ± 24.37 μM, *n* = 388) (*p* = 3.6 × 10^−6^; Fig. 3b).

Similarly, the rates of both sPTB and iPTB were significantly increased from 3.54% and 4.28% in all subjects to 5.47% (*p* = 6.18 × 10^−6^) and 8.49% (*p* = 3.0 × 10^−16^) in NAFLD subjects and 6.14% (*p* = 0.0018) and 17.03% (*p* < 10^−31^) in ICP subjects, respectively (Supplementary Fig. 3a, b and Supplementary Table 3). Correlatively, sTBAs were increased from 4.8 ± 5.3 μM in all subjects to 11.6 ± 24.1 (*p* = 1.3 × 10^−6^) and 12.0 ± 20.7 μM (*p* = 3.0 × 10^−16^) in subjects with sPTB and iPTB among the NAFLD patients (Supplementary Fig. 3a, b). Similarly, sTBAs were markedly elevated to 46.5 ± 44.4 (*p* < 10^−31^) and 41.6 ± 41.9 μM (*p* < 10^−31^) in subjects with sPTB and iPTB, respectively, among the ICP patients (Supplementary Fig. 3a, b). Among the NAFLD subjects, sTBAs were significantly higher in subjects with sPTB (11.57 ± 24.06 μM, *n* = 85; *p* = 10^−4^) and iPTB (11.99 ± 20.68 μM, *n* = 132; *p* = 2.8 × 10^−7^) than in the FTB subjects (6.53 ± 10.28 μM, *n* = 1337) (Supplementary Fig. 3c). Similarly, among the subjects with ICP, sTBAs were significantly elevated from 28.33 ± 24.37 μM in FTB subjects (*n* = 388) to 46.54 ± 44.38 μM in subjects with sPTB (*n* = 31; *p* = 2.0 × 10^−4^) and 41.62 ± 41.92 μM subjects with iPTB (*n* = 86; *p* = 10^−4^) (Supplementary Fig. 3c).

Consistent with liver injuries and increased sTBA levels in patients with NAFLD and ICP, serum AST levels were significantly elevated from 16.67 ± 14.90 U L^−1^ in all the subjects to 28.72 ± 35.45 U L^−1^ in NAFLD (*p* < 10^−31^) and 72.40 ± 85.31 U L^−1^ in ICP subjects (*p* < 10^−31^). Elevated AST levels correlated with increased rates of PTB (Fig. 3c). Among the subjects with NAFLD, AST levels were significantly increased in subjects with PTB (44.23 ± 56.97 U L^−1^) when compared to those with FTB (26.20 ± 34.51 U L^−1^) (*p* = 1.9 × 10^−10^; Fig. 3d). Similarly, higher AST levels were detected in PTB subjects (83.16 ± 91.38 U L^−1^) than those with FTB (69.15 ± 84.22 U L^−1^) among ICP patients without reaching a statistical significance (*p* = 0.12; Fig. 3d). Consistently, among the NAFLD subjects, AST levels were significantly increased from 26.20 ± 34.51 U L^−1^ in FTB subjects to 39.72 ± 53.44 U L^−1^ in sPTB (*p* = 7.8 × 10^−4^) and 47.13 ± 59.14 U L^−1^ in iPTB subjects (*p* = 10^−9^) (Supplementary Fig. 3d). Similarly, among ICP subjects, serum AST levels were elevated in subjects with sPTB (84.58 ± 90.34 U L^−1^) and iPTB (82.65 ± 92.27 U L^−1^) when compared to the FTB subjects (69.15 ± 84.22) without reaching a statistical significance (Supplementary Fig. 3d).

### Estrogen induced cholestasis and PTB in pregnant mice

**Fig. 4:**
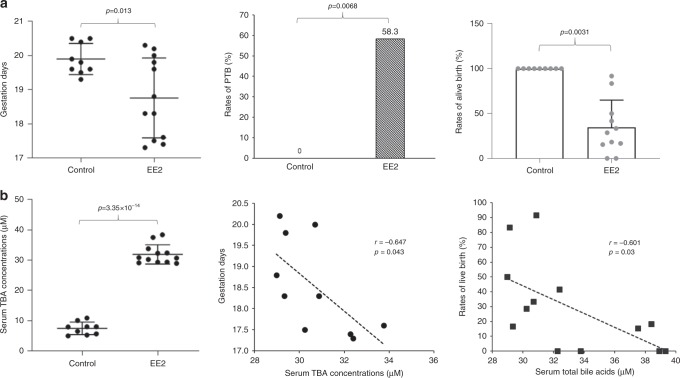
EE2-induced liver injuries, bile acid dysregulation, and PTB in pregnant mice. **a** The gestation days, PTB rates, and rates of live birth in controls (*n* = 9) and EE2-treated pregnant mice (*n* = 12). The data are presented as mean ± SD in the right and left panels. **b** The sTBA levels in controls (*n* = 9) and EE2-treated mice (*n* = 12) and a negative correlation between sTBA concentrations and the gestation days or the rates of live birth. The means and standard errors of the group values were indicated by the long and short lines, respectively. Student’s *t* test for pairwise comparison (two-sided), Chi-squared test for proportion comparisons, and Pearson correlation analysis were applied for statistical analysis. Source data are provided as a Source Data file.

Previous investigations reported that elevated reproductive hormones including estrogens during the late stage of pregnancy contributed to the pathogenesis of ICP^23–25^. Indeed, estrogen-induced cholestasis as animal models for ICP has been well established in rodents^26,27^. However, no studies have been carried out to investigate the effects of estrogen-induced cholestasis on the outcome of pregnancy in rodent models. In this study, we carried out experiments to determine whether estrogen-induced cholestasis and bile acid dysregulation can reproduce the PTB observed in ICP patients. As shown in Fig. 4a, treatment of pregnant mice with synthetic estrogen 17α-ethynylestradiol (EE2) starting at gestation day 16.3 significantly induced PTB. The mean gestation days were significantly decreased from 19.9 ± 0.44 days in control mice (*n* = 9) to 18.76 ± 1.15 days in EE2-treated mice (*n* = 12) (*p* = 0.013). Among the treated mice, 58.3% gave PTB (earlier than 19.1 days) and a large portion of newborns were dead with a huge variation from mouse to mouse (*p* = 0.0068). The rates of live birth ranged from 0% to 91.7% with an average of 34.44 ± 29.78%, which is significantly lower than the control (*p* = 0.0031; Fig. 4a). sTBAs were markedly elevated from 7.49 ± 2.01 μM in control to 31.91 ± 3.12 μM in treated mice (*p* = 3.5 × 10^−14^) and negatively correlated with gestation days (*r* = −0.647, *p* = 0.043) and the rates of live birth (*r* = −0.601, *p* = 0.03) (Fig. 4b). As expected, AST levels were significantly elevated from 17.3 ± 8.5 U L^−1^ in control to 87.3 ± 19.2 U L^−1^ in treated mice (*p* = 5.9 × 10^−9^). The AST levels negatively correlated with the rates of live birth (*r* = −0.678, *p* = 0.015) but not with gestation days (*r* = 0.143, *p* = 0.657) and the sTBA concentrations (*r* = 0.135, *p* = 0.675) (Supplementary Fig. 4a). In addition, no significant correlations were detected between the rates of live birth and the gestation days (*r* = −0.177, *p* = 0.583; Supplementary Fig. 4b).

### Carbon tetrachloride (CCl_4_) induced liver injury and PTB in pregnant mice

Currently, no animal models have been established for studying PTB with liver injuries. In this study, we used CCl_4_ as a challenging agent to induce liver injury and bile acid dysregulation and investigate the effects on PTB in pregnant mice. CCl_4_ is a hepatotoxic agent that is commonly used to induce various hepatic injuries, including NAFLD, fibrosis, and non-alcoholic steatohepatitis.

**Fig. 5:**
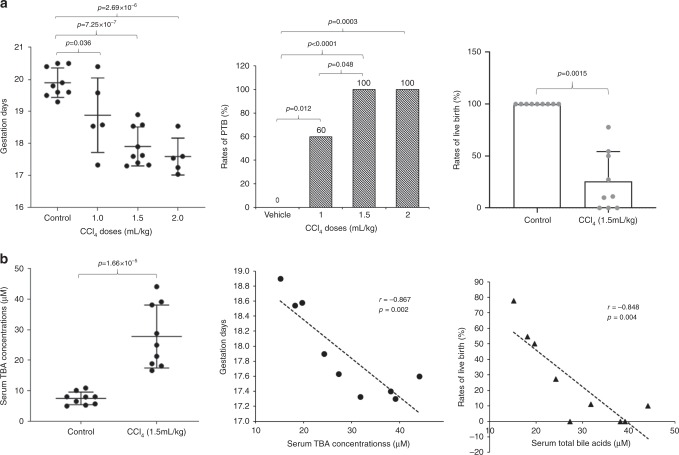
CCl_4_-induced liver injuries, bile acid dysregulation, and PTB in pregnant mice. **a** The gestation days, PTB rates and rates of live birth in controls (*n* = 9) and pregnant mice treated with 1.0 (*n* = 5), 1.5 (*n* = 9), or 2 ml kg^−1^ CCl_4_ (*n* = 5). **b** The sTBA levels in controls (*n* = 9) and mice treated with 1.5 kg ml^−1^ CCl_4_ (*n* = 9) and a negative correlation between sTBA concentrations and gestation days or the rates of live birth. The means and standard errors of the group values were indicated by the long and short lines, respectively. Student’s *t* test for pairwise comparison, one-way ANOVA followed by Tukey post hoc test for multiple group comparison, Chi-squared test for proportion comparisons and Pearson correlation test were applied for statistical analysis. Source data are provided as a Source Data file.

As shown in Fig. 5a, CCl_4_ dose-dependently induced PTB. The mean gestation days were significantly shortened from 19.8 ± 0.5 days in control (*n* = 9) to 18.9 ± 1.1 (*p* = 0.036), 18.1 ± 0.6 (*p* = 7.3 × 10^−7^), and 17.6 ± 0.5 days (*p* = 2.7 × 10^−6^) in mice treated with 1 (*n* = 5), 1.5 (*n* = 9), and 2 ml kg^−1^ CCl_4_ (*n* = 5), respectively. Dosing of 1 ml kg^−1^ CCl_4_ resulted in 60% PTB while 100% mice gave PTB with the doses of 1.5 and 2 ml kg^−1^ CCl_4_. The minimal dose (1.5 ml kg^−1^) that induced 100% PTB was used for subsequent studies, and the mice in this group were further characterized. The rates of live birth were markedly decreased to 26.63 ± 27.82% in mice treated with 1.5 ml kg^−1^ CCl_4_ (*p* = 0.0015; Fig. 5a). sTBAs were significantly elevated from 7.49 ± 2.01 μM in control to 27.78 ± 10.02 μM in treated mice (*p* = 1.7 × 10^−5^) and negatively correlated with the gestation days (*r* = −0.867, *p* = 0.002) as well as the rates of live birth (*r* = −0.848, *p* = 0.004) (Fig. 5b).

As expected, AST concentrations were markedly elevated from 17.3 ± 8.5 U L^−1^ in control to 122.1 ± 22.8 U L^−1^ in CCl_4_-treated mice (*p* = 1.1 × 10^−9^) and positively correlated with sTBA levels (*r* = 0.765, *p* = 0.016). However, no significant correlations were detected between AST levels and the gestation days (*r* = −0.545, *p* = 0.129) nor with the rates of live birth (*r* = −0.49, *p* = 0.181; Supplementary Fig. 5a). It was noted that most of the dead newborns were from the mice having extremely early PTB around gestation day 17.5. Consistently, the rates of live birth positively correlated with the gestation days (*r* = 0.967, *p* < 0.001; Supplementary Fig. 5b).

### Bile acid cholic acid (CA) induced PTB in pregnant mice

**Fig. 6:**
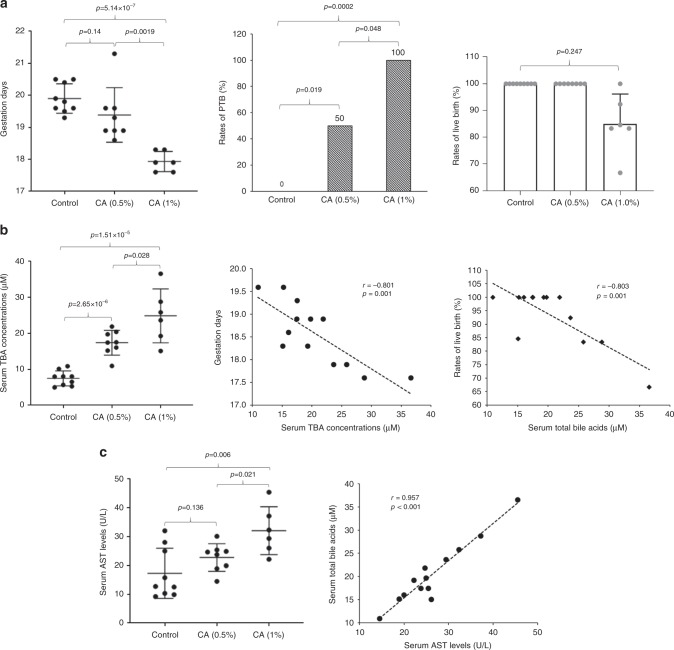
Bile acid CA effectively induced PTB in pregnant mice. **a** The gestation days, PTB rates, and the rates of live birth in control (*n* = 9) and mice treated with a diet containing 0.5% (w/w) (*n* = 8), or 1% CA (*n* = 6). **b** The sTBA levels in controls (*n* = 9) and mice treated with CA diet (*n* = 13) and a negative correlation between the sTBA levels and the gestation days or the rates of live birth. **c** Serum AST levels in controls (*n* = 9) and CA-treated mice (*n* = 13) and a direct correlation between sTBA and AST levels in CA-treated mice. The means and standard errors of the group values were indicated by the long and short lines, respectively. One-way ANOVA, followed by Tukey post hoc test and Chi-squared test for proportion comparisons were applied for statistical analysis. Pearson correlation analysis was applied to determine the correlation coefficient and associated *p* values. Source data are provided as a Source Data file.

The data from pregnant women and mice treated with EE2 or CCl_4_ all demonstrated that sTBA levels positively correlated with the PTB rates. We hypothesized that elevated serum bile acids directly induced PTB. To test the hypothesis, pregnant mice were treated with a diet containing 0.5% or 1% CA starting at gestation day 15.3. As shown in Fig. 6a, CA dose-dependently induced PTB. The average gestation days were decreased from 19.9 ± 0.4 in the control group (*n* = 9) to 19.4 ± 0.8 with a PTB rate of 50% in mice fed with 0.5% CA diet (*n* = 8; *p* = 0.14) and 17.9 ± 0.3 with a PTB rate of 100% in mice fed with 1% CA diet (*n* = 6; *p* = 5.1 × 10^−7^). All the newborns were alive in mice fed with 0.5% CA diet while on average 85.0 ± 10.6% of newborns were alive in the group fed with 1% CA.

As expected, sTBA levels were significantly elevated in the two treatment groups. The average sTBAs were increased from 7.49 ± 2.01 μM in control mice to 17.4 ± 3.3 μM in mice fed with 0.5% CA diet (*p* = 2.7 × 10^−6^) and 24.9 ± 7.2 μM in mice fed with1% CA diet (*p* = 1.5 × 10^−5^) (Fig. 6b). Correlation analysis revealed that sTBAs negatively correlated with the gestation days (*r* = −0.801, *p* = 0.001) and the rates of live birth (*r* = −0.803, *p* = 0.001) (Fig. 6b).

Consistent with CA being a mild bile acid in inducing hepatotoxicity, AST levels were only slightly increased from 17.3 ± 8.5 U L^−1^ in control to 22.8 ± 4.6 (*p* = 0.14) and 32.1 ± 7.9 U L^−1^ (*p* = 0.006) in CA-treated mice (Fig. 6c). It should be noted that the AST levels among the three groups are all within the normal physiological range for mice (from 10 to 45 U L^−1^), indicating that minimal liver injury was induced by CA treatment. Correlation analysis showed that AST levels negatively correlated with gestation days (*r* = −0.835, *p* < 0.001) and the rates of live birth (*r* = −0.892, *p* < 0.001; Supplementary Fig. 6a) while positively correlated with sTBAs (*r* = 0.957, *p* < 0.001; Supplementary Fig. 6a). A direct correlation between the gestation days and rates of live birth was also detected (*r* = 0.774, *p* = 0.002; Supplementary Fig. 6b).

### Elevated bile acids, not liver injury, induced PTB

**Fig. 7:**
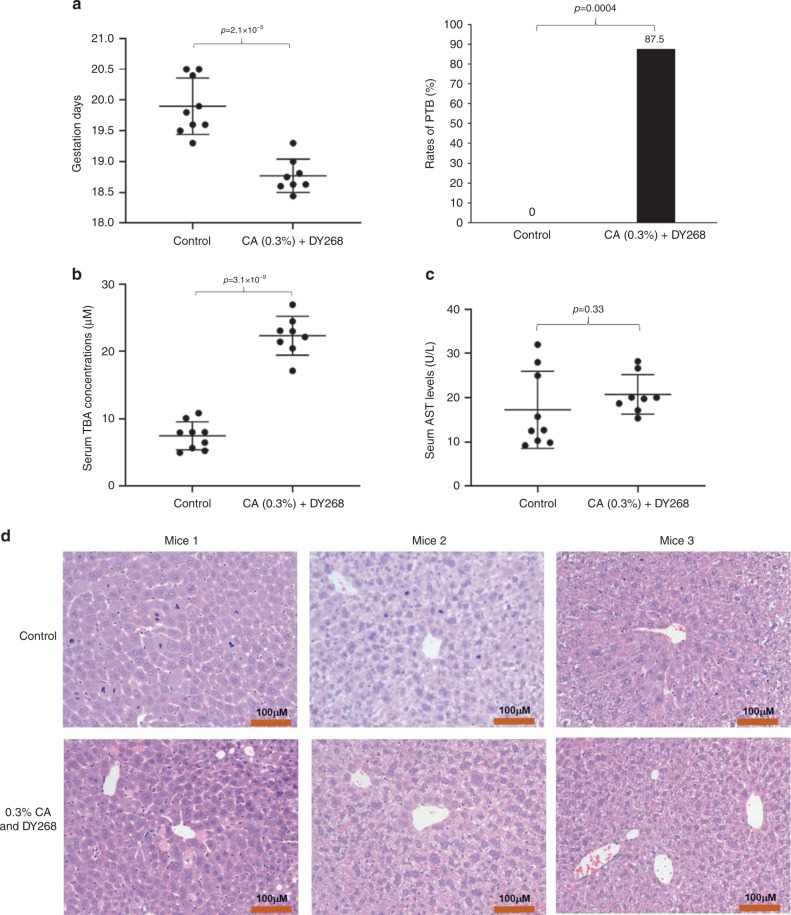
Elevated bile acids, not liver injury, induced PTB. **a** The gestation days and PTB rates in control (*n* = 9) and mice treated with a combination of 0.3% CA and DY268 (*n* = 8). **b** The sTBA concentrations in the control (*n* = 9) and treatment group (*n* = 8). **c** The AST levels in control (*n* = 9) and mice treated with a combination of CA and DY268 (*n* = 8). **d** Representative hepatic histopathology of control and mice treated with CA and DY267. Consistent results were obtained from the control (*n* = 8) and treated mice (*n* = 8). Student’s *t* test for pairwise comparison (two-sided) and Chi-squared test for proportion comparisons were applied for statistical analysis. Source data are provided as a Source Data file.

As presented above, 0.5% CA in diet induced 50% PTB with minimal liver injury as AST levels did not differ from the controls. The data indicated that it was the elevated bile acids, not liver injury, that induced PTB. To further confirm such notion, we carried out additional studies to elevate bile acid levels without inducing liver injury. First, we treated the non-pregnant mice with a reduced dose of CA (0.3%) or FXR antagonist DY268 to determine the effects of the treatments on sTBA and AST levels. It was reasoned that FXR antagonism would increase endogenous bile acid levels through increasing bile acid synthesis and reducing bile acid disposition. As shown in Supplementary Fig. 7a, sTBA levels were significantly elevated in mice treated with 0.3% CA in diet (*n* = 7; *p* = 7.2 × 10^−5^) or DY268 (*n* = 7; *p* = 9.4 × 10^−5^) when compared to the controls (*n* = 9). However, the AST levels were comparable to the control group (*p* = 0.97 or *p* = 0.92; Supplementary Fig. 7b), indicating that no liver injury was induced by either of the treatments. The bile acid levels in mice treated with 0.3% CA in diet or DY268 were elevated to 14.1 ± 2.6 or 13.7 ± 2.4 μM, which may not be high enough to effectively induce PTB based on the findings with the treatment of 0.5% CA in diet (Fig. 6a, b). Therefore, we treated pregnant mice with a combination of 0.3% CA and DY268. As shown in Fig. 7a, the average gestation days were significantly decreased from 19.9 ± 0.4 in the control group (*n* = 9) to 18.3 ± 0.3 in the treatment groups (*n* = 8) (*p* = 2.1 × 10^−5^). A rate of 87.5% PTB (7 out of 8 mice) was reached in the pregnant mice treated with 0.3% CA and DY268 (*p* = 4 × 10^−4^). As expected, sTBA levels were markedly elevated to 22.3 ± 2.8 μM in the treatment group (*p* = 3.1 × 10^−9^; Fig. 7b), which is significantly higher than the sTBA levels in mice treated with 0.3% CA or DY268 alone (Supplementary Fig. 7a). On the other hand, AST levels were comparable to the control group (*p* = 0.33; Fig. 7c). Histopathology examination confirmed no liver injury (Fig. 7d). Consistent with the findings from pregnant mice treated with 0.5% (Fig. 6a), all the newborns were alive. The data thus firmly established that it was the elevated bile acids, not liver injury itself, that induced PTB.

### Prevention of PTB by restoring bile acid homeostasis

**Fig. 8:**
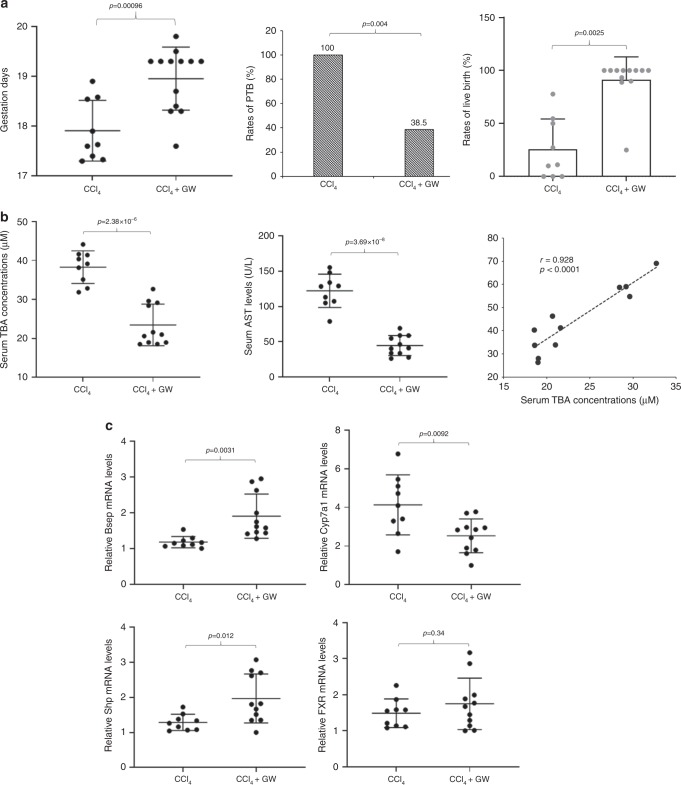
Prevention of PTB by restoring bile acid homeostasis through FXR activation in pregnant mice. **a** The gestation days, PTB rates, and the rates of live birth in mice treated with CCl_4_ alone (1.5 ml kg^−1^) (*n* = 9) or a combination of CCl_4_ (1.5 ml kg^−1^) and FXR agonist GW4064 (50 mg kg^−1^) (*n* = 13). **b** The sTBA and AST levels in mice treated with CCl_4_ alone (*n* = 9) or a combination of CCl_4_ and GW4064 (*n* = 11) and a positive correlation between sTBA and AST levels. **c** The relative expression levels of mRNAs for *Bsep*, *Cyp7a1*, *Shp*, and *Fxr* in mice treated with CCl_4_ (*n* = 9) or a combination of CCl_4_ and GW4064 (*n* = 11). The means and standard errors of the group values were indicated by the long and short lines, respectively. Student’s *t* test for pairwise comparisons (two-sided) and Chi-squared test for proportion comparisons were applied for statistical analysis. Pearson correlation analysis was applied to determine the correlation coefficient and associated *p* values. Source data are provided as a Source Data file.

FXR is the master regulator of bile acid homeostasis. Activation of FXR by bile acids results in reduced bile acid synthesis and enhances bile acid disposition^28,29^. We hypothesized that PTB should be prevented by restoring bile acid homeostasis through FXR activation. To test the hypothesis, pregnant mice were treated with CCl_4_ alone or a combination of CCl_4_ and a synthetic FXR agonist GW 4064. As shown in Fig. 8a, GW4064 significantly increased the gestation days from 17.9 ± 0.59 in mice treated with CCl_4_ (*n* = 9) to 19.1 ± 0.76 in mice treated with both CCl_4_ and GW4064 (*n* = 13) (*p* = 9.6 × 10^−4^). The PTB rates were markedly decreased from 100% to 38.5% (*p* = 0.04) and the rates of live birth were dramatically improved from 25.6 ± 27.8% to 91.4 ± 20.9% (*p* = 0.0025). As expected, sTBAs were significantly reduced from 38.3 ± 4.1 to 23.8 ± 5.5 μM (*p* = 2.4 × 10^−6^). Similar to sTBA, AST levels were significantly decreased from 122.1 ± 22.8 to 43 ± 15.3 U L^−1^ (*p* = 3.7 × 10^−8^) and positively correlated with sTBA concentrations (*r* = 0.928, *p* < 0.0001; Fig. 8b). sTBA concentrations negatively correlated with the gestation days (*r* = −0.775, *p* = 0.005) but not with the rates of live birth (Supplementary Fig. 8a). Serum AST levels negatively correlated with both the gestation days (*r* = −0.725, *p* = 0.012) and the rates of live birth (*r* = −0.66, *p* = 0.038; Supplementary Fig. 8b). A direct correlation between the gestation days and the rates of live birth was also detected (*r* = 0.648, *p* = 0.043; Supplementary Fig. 8c).

As the transporter responsible for the rate-limiting biliary secretion of bile acids in the enterohepatic circulation, the expression of bile salt export pump (*Bsep*) was significantly upregulated by GW4064 (*p* = 0.0031; Fig. 8c), indicating enhanced biliary bile acid disposition. On the other hand, cholesterol 7α-hydroxylase (*Cyp7a1*), a rate-limiting enzyme for bile acid synthesis, was significantly downregulated (*p* = 0.0092), suggesting reduced bile acid synthesis. Consistently, the expression of small heterodimer partner (*Shp*), a repressor for Cyp7a1 expression, was significantly increased (*p* = 0.012). On the other hand, GW4064 treatment had limited effects on *Fxr* expression (*p* = 0.34; Fig. 8c).

## Discussion

The underlying mechanisms by which PTB is induced are complex and multi-factorial^8,9^. Bile acids are recently recognized as signaling molecules to regulate a variety of cellular and metabolic activities^14–16^. In an early study to investigate the effects of bile acids on the fetus complications, direct intravenous infusion of CA into fetal lambs resulted in early delivery^30^. However, the cause of premature labor in the ewes with fetuses infused with CA was not evident. In this study, we found that sTBA concentrations in the pregnant women were directly correlated with the rates of PTB including sPTB and iPTB regardless of the characteristics of the pregnant women, such as maternal age and BMI or the etiologies of liver injuries, such as NAFLD and ICP. Our data are in line with the findings from previous reports that sTBA levels were correlated with the risks for PTB in ICP patients^17–19^. In this study, we further demonstrated that bile acid CA effectively and dose-dependently induced PTB in pregnant mice. The findings thus etiologically link elevated bile acids to PTB. Consistent with our finding, myometrium isolated from pregnant women with ICP was more sensitive to oxytocin than the myometrium from normal pregnant women, which may be due to the upregulation of oxytocin receptor expression in myometrium by bile acids^31,32^. Therefore, dysregulation of bile acids due to liver disorders or other pathological conditions is a previously unrecognized risk factor for PTB. sTBA concentrations may represent a potential predictive marker for PTB.

The liver is the organ responsible for nutritional and energy metabolism. As a physiological adaption, liver size is significantly enlarged during the late stage of pregnancy to meet the nutritional and metabolic demands of the mother and growing fetus^33–35^. Under pathological conditions with dysregulated bile acids due to liver diseases or others, elevated bile acids may act as a message of compromised liver to communicate with and regulate other organs of the body. When the energy demands from the growing fetus and mother cannot be met by the compromised liver, termination of the pregnancy may become the option for the mother to survive. Therefore, early termination of pregnancy or PTB may be considered as a protective or adaptive mechanism for a pregnant woman to survive when liver function is compromised^36,37^. Consistently, similar to sTBA, serum AST, ALT, TBiL and GGT levels, which commonly serve as liver function or damage markers, directly correlated with the PTB rates in both human (Figs. 1d and 3c, Supplementary Figs. 1e, f and 2a, c) and mice (Supplementary Figs. 4a, 5a, 6a, and 8b). In addition, pregnant women with various liver disorders have increased risk for PTB^17–19,38–45^.

Advanced maternal age and being overweight/obese have been associated with increased risk for PTB^20–27^. However, the underlying mechanisms are unknown. In this study, we found that sTBA concentrations were gradually elevated as the maternal age advanced (Fig. 2b) and BMI increased (Fig. 2d). More importantly, positive correlations between elevated sTBAs and increased PTB rates were detected in subjects with advanced maternal age and overweight or obese (Fig. 2b, d). Therefore, elevated circulating bile acids may directly contribute to the increased risk for PTB in those subjects. Consistent with our finding, previous clinical studies with non-pregnant subjects showed that serum bile acids were significantly elevated in obese subjects^46–48^. Currently, advanced age and overweight/obesity in reproductive women are on the rise, which may contribute to the current uptrend of PTB rate^7^. Modulating bile acid homeostasis may be of clinical significance for reducing PTB in age-advanced and overweight/obese subjects.

Among the liver disorders, NAFLD is considered the most common disease^49–51^. Indeed, NAFLD is the commonest cause for liver dysfunction in our study cohort, with 4.2% of the pregnant women being diagnosed with NAFLD. Similar to the subjects with ICP in this study and previous reports^17–19^, NAFLD patients also had increased PTB rates (Fig. 3a). The finding is consistent with a previous study reporting that the risk for PTB increased 2.5-fold in subjects with NAFLD^42^. However, the underlying mechanism is largely unknown. In this study, we found that sTBAs were also significantly elevated in NAFLD subjects (Fig. 3a) and thus may contribute to the induction of PTB in NAFLD subjects. Dysregulation of bile acids in subjects with NAFLD has long been recognized in non-pregnant subjects. A large body of evidence from clinical as well as preclinical studies demonstrate that sTBA concentrations were largely elevated in subjects with NAFLD^52^. Restoring bile acid homeostasis may represent a strategy to reduce the risk for PTB in pregnant women with NAFLD and ICP.

Elevated estrogens during late stages of pregnancy play an important role in the pathogenesis of ICP^53–55^. On the other hand, CCl_4_ has been widely used to induce various hepatic injuries in animals, including NAFLD, fibrosis, and non-alcoholic steatohepatitis^56–58^. In this study, we successfully reproduced PTB in pregnant mice with liver injuries induced by EE2 or CCl_4_. It was noted that high percentages of the newborns were dead in both EE2- and CCl_4_-induced PTB models. However, the underlying mechanisms may differ. In the CCl_4_ model, the rates of live birth negatively correlated with the gestation days (Supplementary Fig. 5b) with majority of dead births from the pregnant mice with extremely early gestation days (17.5 days). The finding is consistent with higher fetal mortality rates in pregnant women with extreme early PTB^59,60^. However, in the EE2 model, the rates of live birth did not directly correlate with the gestation days (Supplementary Fig. 4b), indicating that estrogen-induced cholestasis may have direct adverse effects on the fetuses. In patients with ICP, a high percentage of PTB is iatrogenic (73.5%; Supplementary Fig. 3a, b), which are mainly due to fetal stresses and complications. In addition, increased stillbirths were also reported in pregnant women with ICP^17–19^. The high rates of dead births observed in the EE2 model may represent the sum of iPTB and stillbirth in pregnant women with ICP. It should be noted that the high dose of EE2 (25 mg kg^−^^1^) used in this study may also contribute to the high rates of dead birth. Taken together, EE2-induced PTB may represent a good mouse model to study the pathogenesis of iPTB and stillbirth in pregnant women with ICP, while CCl_4_-induced PTB may represent a good mouse model to study PTB associated with liver disorders or dysregulation of bile acids other than ICP.

In this study, bile acid CA induced PTB as effectively as, if not more effectively than, EE2 and CCl_4_ treatments (Figs. 4a, 5a, 6a, and 7a). Consistent with CA being a mild bile acid in inducing hepatotoxicity^61^, minimal or no liver injury was detected in mice treated with 0.5% CA or a combination of 0.3% CA and FXR antagonist DY268 as the AST levels were comparable to the control group (Figs. 6c and 7c) and histopathology examination revealed no hepatic toxicity (Fig. 7d). The data thus demonstrate that it is the elevated bile acids, not liver injury itself, that induces PTB. Distinct from the EE2, CCl_4_ and other reported PTB mouse models^62^, which have low rates of live birth (34.4 ± 29.8 for EE2 and 25.6 ± 28.7 for CCl_4_ model), majority of the newborns from CA-induced PTB were alive with a rate of live birth 85.0 ± 10.6%, 100%, and 100% in mice treated with 1% CA, 0.5% CA, and a combination of 0.3% CA and DY268, respectively. Therefore, CA-induced PTB represents a good model for studying the developmental consequences of the newborns. In addition, the findings also indicate that elevated bile acids are the trigger for inducing PTB while liver injuries induced by EE2, CCl_4_, or other factors may contribute to the low rates of live birth. Therefore, sTBA can potentially serve as a better marker for predicting PTB while other liver injury tests, such as AST, can potentially serve as better predictors for the health status of the fetus. Our findings indicated that reducing bile acids potentially prevent or delay PTB while promoting liver repairing or improving liver function may increase the survival rate of the newborns in pregnant women with liver disorders, including cholestasis. Considering the fact that some subjects had elevated bile acids but were protected from PTB, other factors such as individual variations in signaling pathways that mediate bile acid-induced PTB exist and remain to be identified.

Current interventions for preventing or delaying PTB did not achieve consistent improvement^10,11^. New mechanism-based interventions are in need. In this study, we demonstrated that restoring the bile acid homeostasis by FXR activation significantly reduced CCl_4_-induced PTB and dramatically improved the survival rates of the newborns. The finding holds a great promise for developing new therapies to prevent or delay PTB for pregnant women at high risk for PTB with dysregulated bile acids due to liver disorders or other conditions.

## Methods

### Study cohort

A prospective cohort was recruited at the Nantong Maternal and Child Health Hospital (NMCH) affiliated to Nantong University, China. The medical history and supporting clinical and laboratory information were collected at baseline. All pregnant women aged 18–50 years were screened. Exclusion criteria were described previously^41^. Data on maternal demographic characteristics were collected from questionnaires completed by women at the first antenatal visit^41^. A total of 44,906 pregnant women were screened during their first trimester of pregnancy at the NMCH. Of these, 6242 were excluded owing to: multiple pregnancy in 1518 subjects, other infection in 2496 subjects, concurrent medical complications (diabetes, hypertension, or heart diseases) in 841 subjects, and spontaneous or induced abortion in 2787 subjects. Thereafter, 37,264 pregnant women were recruited in this study. Most of the enrolled subjects received at least three health examinations and were followed up until delivery. Five hundred and nine subjects were excluded because of lost to follow-up (292 subjects) or incomplete data (217 subjects). Finally, after exlusion, a total of 36,755 pregnant women were included in the study. We tried to include as many participants as possible and did not exclude participants if they fulfilled the inclusion criteria and follow-up. Self-selection bias was prevented by the blinding of investigators who collected the data and those who analyzed the data. Diagnoses of NAFLD and ICP were made according to relevant guidelines^63,64^. The primary outcome was PTB defined as earlier than 37 weeks of gestation. The PTB was further categorized into spontaneous (either following onset of contractions or spontaneously ruptured membranes) and iatrogenic (medically indicated labor due to maternal or fetal reasons) PTB^65^. The cohort study was performed according to the Declaration of Helsinki and approved by the Institutional Review Board of NMCH. Written informed consents were obtained from all participants. The study was retrospectively registered to the Research Registry (https://www.researchregistry.com/) with an identification number 5434.

### Chemicals and reagents

CA, CCl_4_, GW4064, olive oil, and EE2 were purchased from Sigma-Aldrich. 1,2-Propanediol and corn oil were purchased from Acros Organics. Mouse TBA kits were purchased from Crystal Chem. Mouse AST Kit was purchased from Biovision.

### Mouse models for PTB with liver injuries

Timed pregnancy of CD-1 mice was achieved by mating overnight. Next day when the male mice were removed is considered gestation day 0. EE2 was administered subcutaneously once daily at a dose of 25 mg kg^−1^ in a solution of 80% 1,2-propanediol and 0.15% NaCl on gestation day 16.3. The treatment was continued until labor. Single injection of CCl_4_ was administered intraperitoneally at a dose of 1, 1.5, or 2 ml kg^−1^ in olive oil (40% v/v) on gestation day 16.3. Upon initiation of the treatment, pregnant mice were closely monitored for labor. The rates of live birth were recorded. The mice were euthanized after the labor, and blood and tissues samples were collected for analyses. All the mouse studies were approved by the Institutional Animal Care and Use Committee of the University of Rhode Island. All mice were housed in ventilated cages in a 12-h light–dark cycle (starting at 6:00 a.m. and 6:00 p.m.), with controlled room temperature (20–22 °C) and relative humidity (40%). Mice had access to food and water ad libitum.

### Treatment of pregnant mice with bile acid CA and FXR antagonist DY268

Treatment with 0.5% (w/w) or 1% CA in a regular chow diet was initiated at gestation day 15.3 and continued until labor. Treatment with a combination of 0.3% CA (in diet) and FXR antagonist DY268 (20 mg kg^−1^, oral gavage, twice a day) was also initiated at gestation day 15.3 and continued until labor. Pregnant mice were closely monitored for labor after initiation of the treatment. The rates of live birth were recorded. The mice were euthanized after the labor. Blood and tissues samples were collected for analyses. For the study with non-pregnant mice, mice were treated with 0.3% CA in diet or DY268 (20 mg kg^−1^, oral gavage, twice a day) for 3 days. At the end of the study, mice were euthanized, and blood samples were collected for sTBA and AST analyses.

### Treatment of pregnant mice with a combination of CCl_4_ and FXR agonist GW4064

Pregnant mice were treated with FXR agonist GW4064 on gestation day 15.3 through oral gavage twice a day with a dose of 50 mg kg^−1^ in corn oil and the treatment continued till labor. Twenty-four hours after the first treatment of GW4064, the pregnant mice were treated with CCl_4_ at a dose of 1.5 ml kg^−1^ in 40% olive oil through intraperitoneal injection at gestation day 16.3. The mice were closely monitored for labor after CCl_4_ treatment. The gestation days and rates of live birth were recorded. The mice were euthanized after the labor. Blood and tissues samples were collected for analyses.

### TaqMan quantitative real-time PCR

Total RNA was isolated with RNA-Bee regent (Tel-Test, Friendswood, TX). cDNA was synthesized with 1 μg of total RNA and random primers in a total volume of 25 μl. The reactions were incubated initially at 25 °C for 10 min and then at 50 °C for 50 min, followed by inactivation of the reaction at 70 °C for 10 min. The cDNAs were then diluted five times with water and subjected to real-time PCR using TaqMan Gene Expression Assay (Applied Biosystems, Foster City, CA). The real-time PCR was performed using TaqMan Universal PCR Master Mix (Applied Biosystems) in a total volume of 20 μl containing 10 μl of universal PCR master mix, 1 μl of gene-specific TaqMan assay mixture, and 5 μl of cDNA templates. Cycling profile was 50 °C for 2 min, 95 °C for 10 min, followed by 40 cycles of 15 s at 95 °C and 1 min at 60 °C, as recommended by the manufacturer. Amplification and quantification were done with the Applied Biosystems 7500 Real-Time PCR System. The TaqMan assay probes for *Fxr* (assay ID: Mm00436425_m1), *Shp* (assay ID: Mm00442278_m1), *Cyp7a1* (assay ID: Mm00484150_m1), *Bsep* (assay ID: Mm00445168_m1), and glyceraldehyde 3-phosphate dehydrogenase (*Gapdh*) (assay ID: Mm99999915_g1) were purchased from Applied Biosystems. Transcript levels of *Fxr*, *Shp*, *Cyp7a1*, and *Bsep* were normalized against the GAPDH levels. Validated TaqMan PCR probes and master mixtures were obtained from Applied Biosystems.

### Liver function tests

All recruited pregnant women had baseline liver function tests performed at the first antenatal visit at gestation weeks 12–14, followed by at least one subsequent testing at gestation weeks 24–28. Additional testing was requested as clinically appropriate at gestation weeks 32–36. Serum samples were collected from pregnant women after fasting for at least 8 h. sTBA, AST, ALT, GGT, and TBiL levels were measured using an automatic biochemical analyzer (AU2700, Olympus, Japan). The highest values among the 2–3 tests for each individual subjects were used for the data analyses. Mouse sTBAs were quantified with the mouse TBA Kit from Crystal Chem. Mouse AST assays were carried out with the Mouse AST Kit from Biovision.

### Histopathology

Fresh liver tissues were collected and fixed with 10% formalin immediately after euthanization of the mice. After fixation in 10% formalin solution for at least 24 h, the liver tissue samples were delivered to the Molecular Pathology Core at Brown University for paraffin embedding, sectioning, hematoxylin and eosin staining, and histopathology examination by a pathologist. The samples were randomized and blinded when they were delivered to the core. After the histopathology report was provided to us, we decoded the samples and presented the data.

### Statistics

Student’s *t* test was applied to pairwise comparison for normally distributed data. One-way analysis of variance was applied to analyze data with multiple groups, followed by Tukey post hoc test for multiple comparisons. Chi-squared test was applied for proportion comparisons. Pearson correlation analysis was applied to determine the correlation coefficient (*r*) and associated *p* values. Poisson regression model was used to estimate the RR with 95% CI. A *p* value of ≤0.05 was considered statistically significant. IBM SPSS Statistics 25, SAS, and Prism version 8 GraphPad software were used for statistical analyses.

### Reporting summary

Further information on research design is available in the Nature Research Reporting Summary linked to this article.

## Supplementary information

Supplementary Information

Reporting Summary

## Data Availability

All the relevant data that support the findings of this study are available from the corresponding author upon request. The source data underlying Figs. 1a–e, 2a–d, 3a–d, 4a, b, 5a, b, 6a–c, 7a–c, 8a–c and Supplementary Figs. 1a–k, 2a–d, 3a–d, 4a, b, 5a, b, 6a, b, 7a, b, 8a–c are provided as a Source Data file.

## Source Data

Source Data
