# Supplementary material for: Dysregulation of bile acids increases the risk for preterm birth in pregnant women
Source: Nat Commun. 2020 Apr 30;11:2111. doi: 10.1038/s41467-020-15923-4 (PMC7193585; doi:10.1038/s41467-020-15923-4)
Supplement: Supplementary file 1 — Supplementary Information [file 41467_2020_15923_MOESM1_ESM.pdf]

Dysregulation of bile acids increased the risk for preterm birth in pregnant women

Sangmin You, Ai-Min Cui2, Syed F. Hashmi, et al.

Supplementary Fig. 1

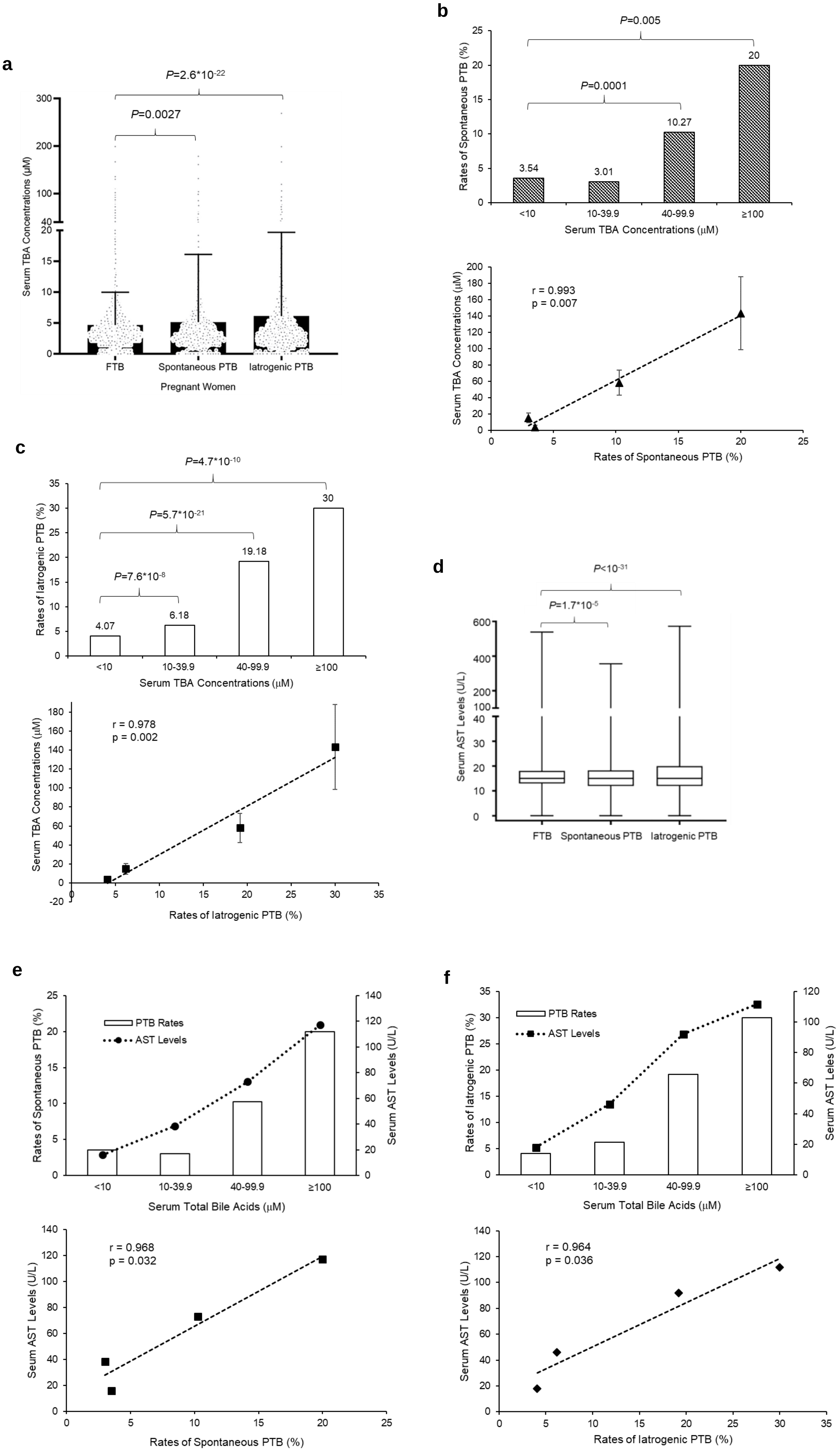

Supplementary Fig. 1

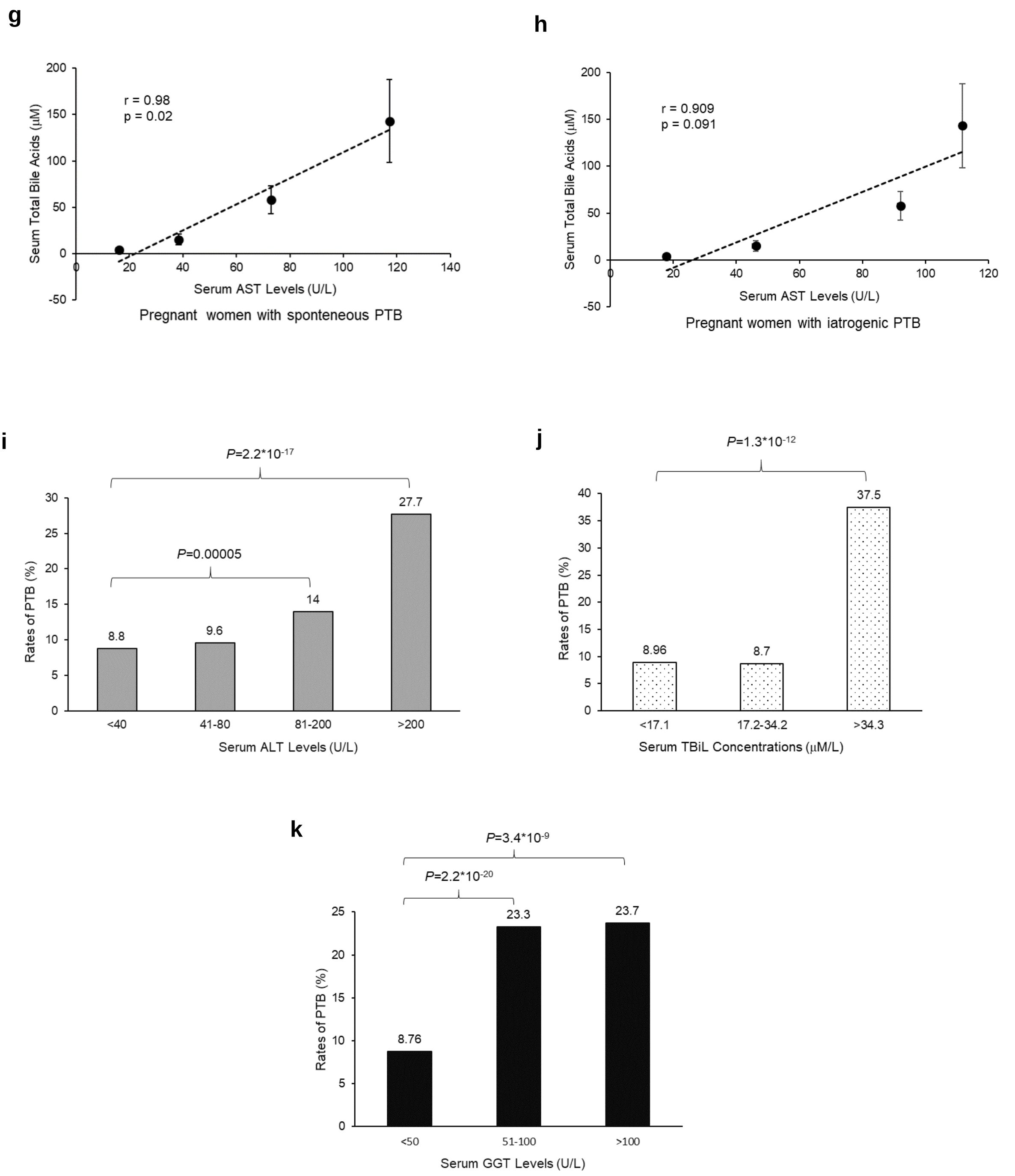

Supplementary Fig. 1. Elevated sTBA and AST levels in pregnant women with sPTB and iPTB. (**a**) the sTBA levels in subjects with FTB (n=33,880) and subjects with sPTB (n=1,302) or iPTB (n=1,573). The box and whisker plots show the maximum, minimum data points, median value and 75th and 25th quartile. (**b**) as the sTBA concentrations elevated from <10 (n=34,227) to 10-39.9 (n=2,362), 40-99.9 (n=146) and ≥100 μM (n=20), the rates of sPTB increased. The sTBA levels directly correlated with rates of sPTB. The data points for serum TBA levels are presented as mean ± SD in the bottom panel. (**c**) As the sTBA levels elevated from <10 (n=34,227) to 10-39.9 (n=2,362), 40-99.9 (n=146) and ≥100 μM (n=20), the rates of iPTB increased. The sTBA levels directly correlated with the rates of iPTB. The data points for serum TBA levels are presented as mean ± SD in the bottom panel. (**d**) serum AST levels in subjects with FTB (n=33,880), sPTB (n=1,302) and iPTB (n=1,573). The box and whisker plots show the maximum, minimum data points, median value and 75th and 25th quartile. (**e**) as the AST levels elevated from 0-40 (n=35,360) to 41-80 (n=1,046), 81-200 (n=284) and > 200 U/L (n=65), both sTBA levels and the rates of sPTB increased. The AST levels positively correlated with the rates of sPTB. (**f**) as the AST levels elevated from 0-40 (n=35,360) to 41-80 (n=1,046), 81-200 (n=284) and > 200 U/L (n=65), both sTBA levels and the rates of iPTB increased. The AST levels directly correlated with the rates of iPTB. (**g**) the AST levels positively correlated with sTBA in pregnant women with sPTB or (**h**) iPTB. The data points for serum TBA levels in both (g) and (h) are presented as mean ± SD in the bottom panel. (**i**) as the ALT levels elevated from 0-40 (n=34,393) to 41-80 (n=1,619), 81-200 (n=613) and >200 U/L (n=130), the rates of PTB increased, (**j**) as the TBiL levels increased from 0-17.1 (n=35,929) to 17.2-34.2 (n=770) and >34.2 μM/L (n=56), the rates of PTB increased and (**k**) as the GTT levels elevated from 0-50 (n=36,148) to 51-100 (n=489) and > 100 U/L (n=118), the rates of PTB increased. One-way ANOVA, followed by Tukey post-hoc test and Pearson correlation test were applied for statistical analysis. Source data are provided as a Source Data file.

Supplementary Fig. 2

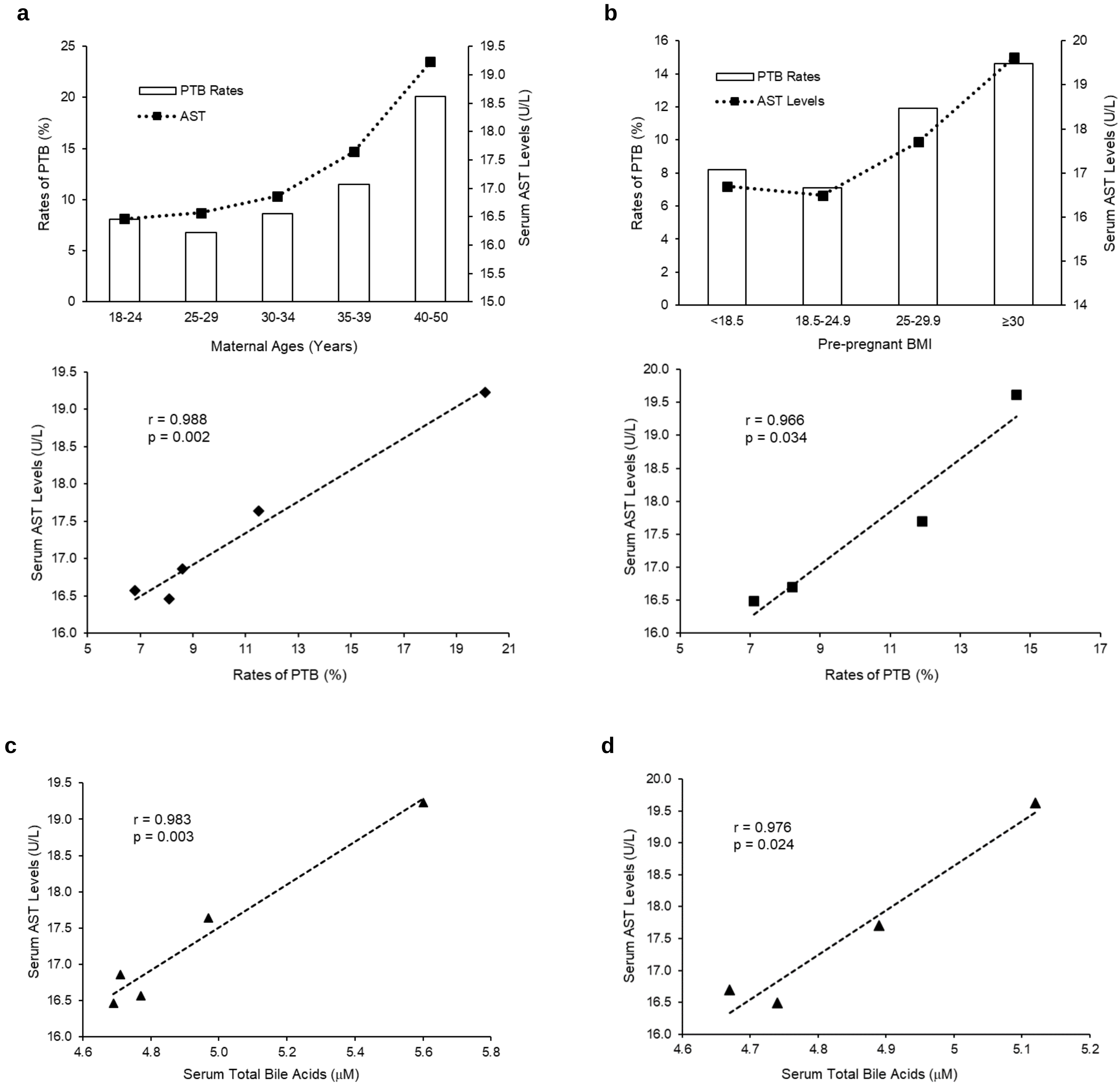

Supplementary Fig. 2. Serum AST levels were elevated in pregnant women with advanced maternal age or high BMIs who had increased risk for PTB. (**a**) serum AST levels in the five age groups, 18-24 (n=9,000), 25-29 (n=19,289), 30-34 (n=6,122), 35-39 (n=2,025) and 40-50 (n=319), and a positive correlation between serum AST levels and PTB rates among the five age groups of pregnant women. (**b**) the serum AST levels in the four BMI groups, <18.5 (n=2,973), 18.5-24.9 (n=29,004), 25-29.9 (n=4,593) and ≥30 (n=185), and a positive correlation between AST levels and the PTB rates was detected among the four BMI groups. (**c**) a direct correlation between sTBA and AST levels was detected among the five age groups of pregnant women with the sample sizes described in (a). (**d**) the serum AST and sTBA levels were directly correlated each other among the four BMI groups with the sample sizes described in (b). Pearson correlation analysis was applied to determine the correlation coefficient and associated p values. Source data are provided as a Source Data file.

Supplementary Fig. 3

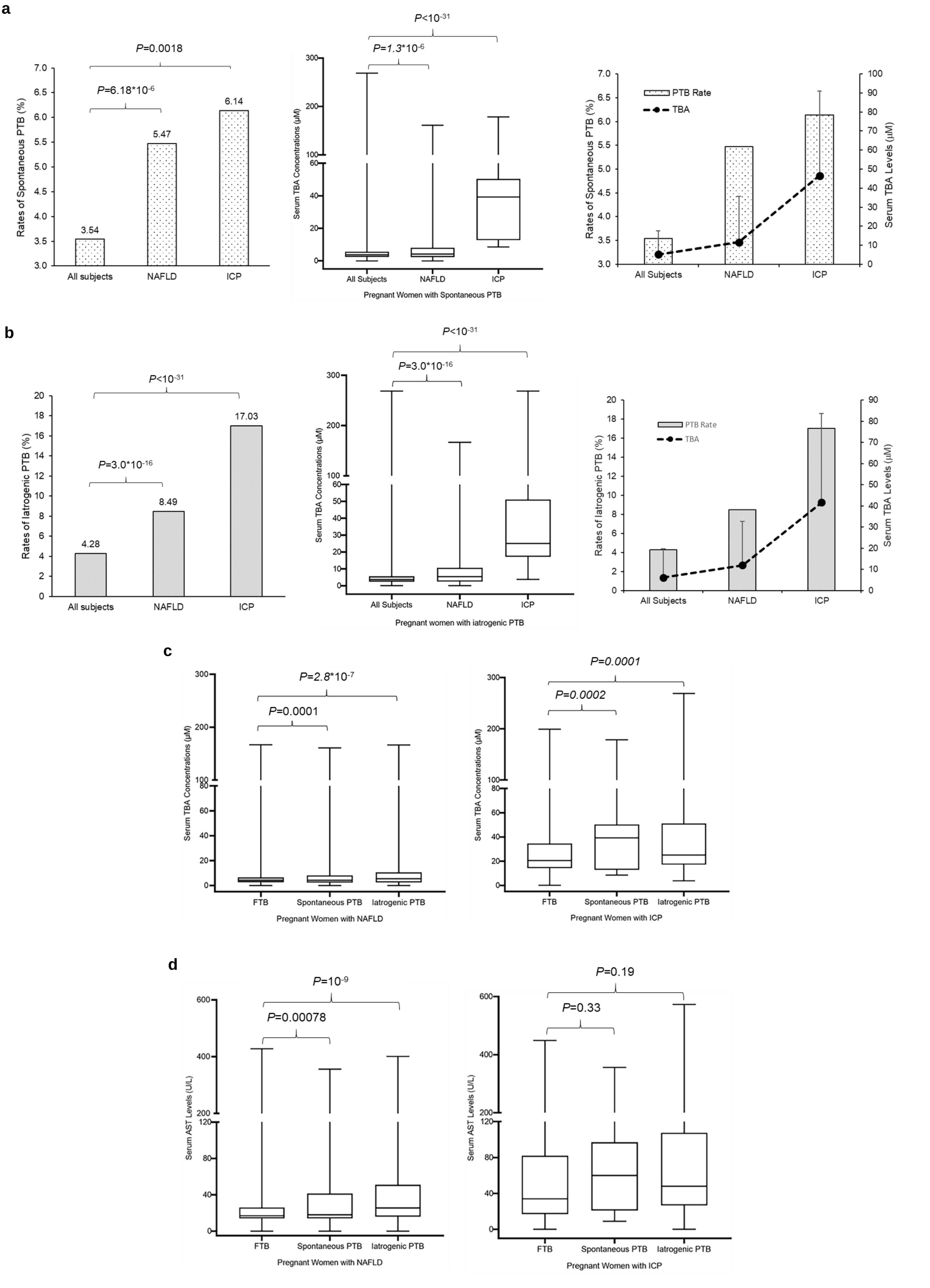

Supplementary Fig. 3. Serum TBA and AST levels were elevated in subjects with sPTB and iPTB among the pregnant women with NAFLD or ICP. (**a**) the rates of sPTB in all pregnant women (n=1,302 among 36,755 subjects) and pregnant women with NALFD (n=85 among 1,554 subjects) and ICP (n=31 among 505 subjects). The sTBA levels were elevated in the sPTB subjects with NAFLD or ICP when compared with the sTBA levels in all subjects. The sTBA levels were correlated with the rates of sPTB. The box and whisker plots in the middle panel show the maximum, minimum data points, median value and 75th and 25th quartile. The data points for serum TBA levels are presented as mean ± SD in the right panel. (**b**) the iPTB rates in all pregnant women (n=1,573 among 36,755 subjects) and subjects with NALFD (n=132 among 1,554 subjects) and ICP (n=86 among 505 subjects). The levels of sTBA were elevated in the iPTB subjects diagnosed with NAFLD or ICP when compared to the bile acid levels in all subjects. The sTBA levels correlated with the rates of iPTB. The box and whisker plots in the middle panel show the maximum, minimum data points, median value and 75th and 25th quartile. The data points for serum TBA levels are presented as mean ± SD in the right panel. (**c**) among the NAFLD pregnant women (n=1,554), the sTBA levels were significantly higher in subjects with sPTB (n=85) and iPTB (n=132) than the subjects with FTB (n=1,337). Among the ICP subjects (n=505), serum TBA levels were significantly elevated in subjects with sPTB (n=31) and iPTB (n=86) when compared to the levels in subjects with FTB (n=388). The box and whisker plots in both panels show the maximum, minimum data points, median value and 75th and 25th quartile. (**d**) the AST levels in subjects with FTB (n=1,337 for NAFLD and n=388 for OCP), sPTB (n=85 for NAFLD and n=31 for ICP) and iPTB (n=132 for NAFLD and n=86 for ICP) among the pregnant women with NAFLD or ICP. The box and whisker plots in both panels show the maximum, minimum data points, median value and 75th and 25th quartile. One-way ANOVA, followed by Tukey post-hoc test, was applied for multiple group comparison. Source data are provided as a Source Data file.

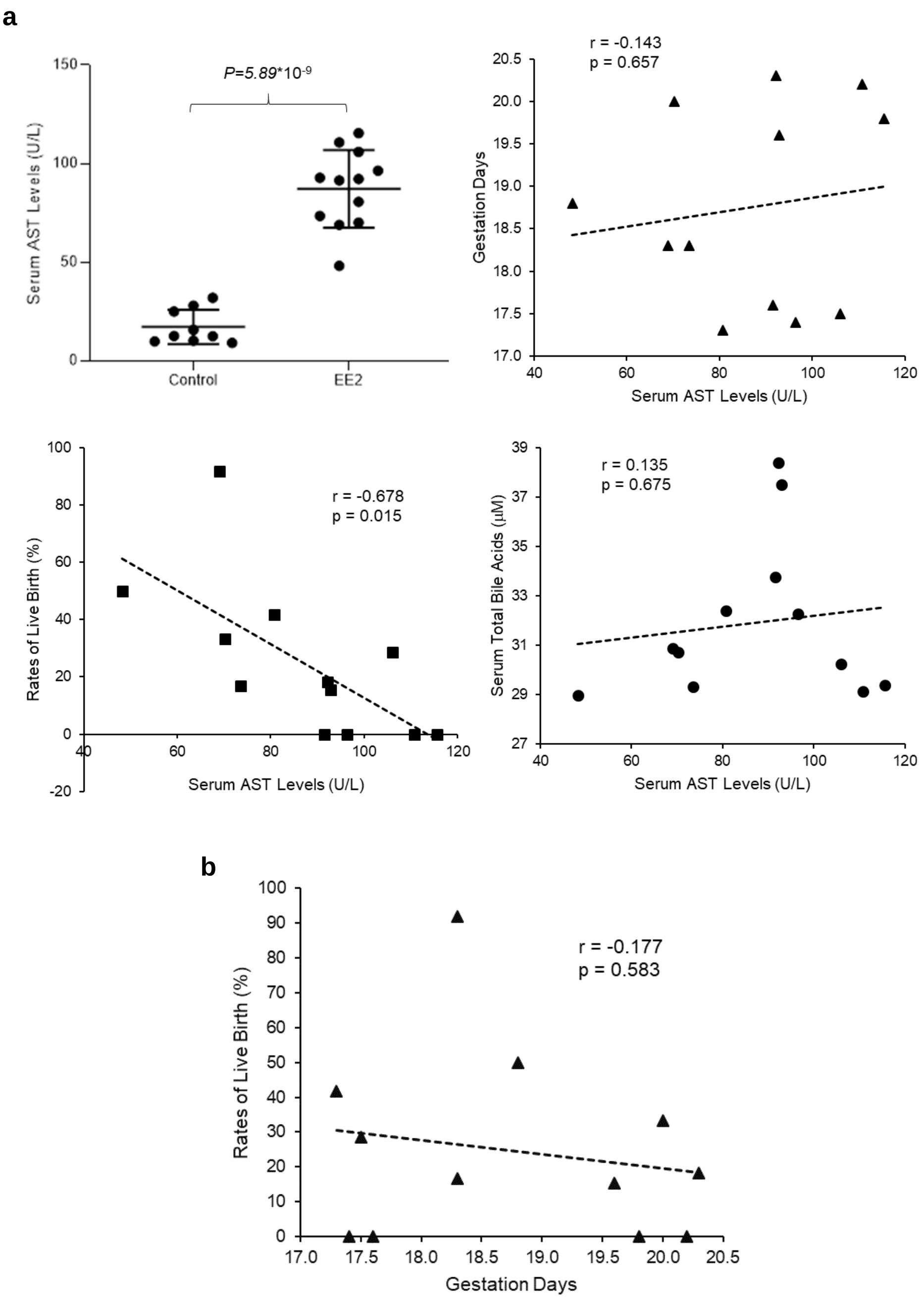

Supplementary Fig. 4. Elevated serum AST levels in EE2-inudced liver injuries and PTB. (**a**) the serum AST levels in control (n=9) and EE2-treated mice (n=12), and correlation analyses between serum AST levels and gestation days, rates of live birth or sTBA levels. The data points for serum AST levels are presented as mean ± SD in the upper left panel. (**b**) no correlation between the gestation days and the rates of live birth in EE2-treated mice was detected. The means and standard errors of the group values were indicated by the long and short lines, respectively. Student's t-test for pairwise comparison (two-sided) and Pearson correlation test were applied for statistical analysis. Source data are provided as a Source Data file.

a

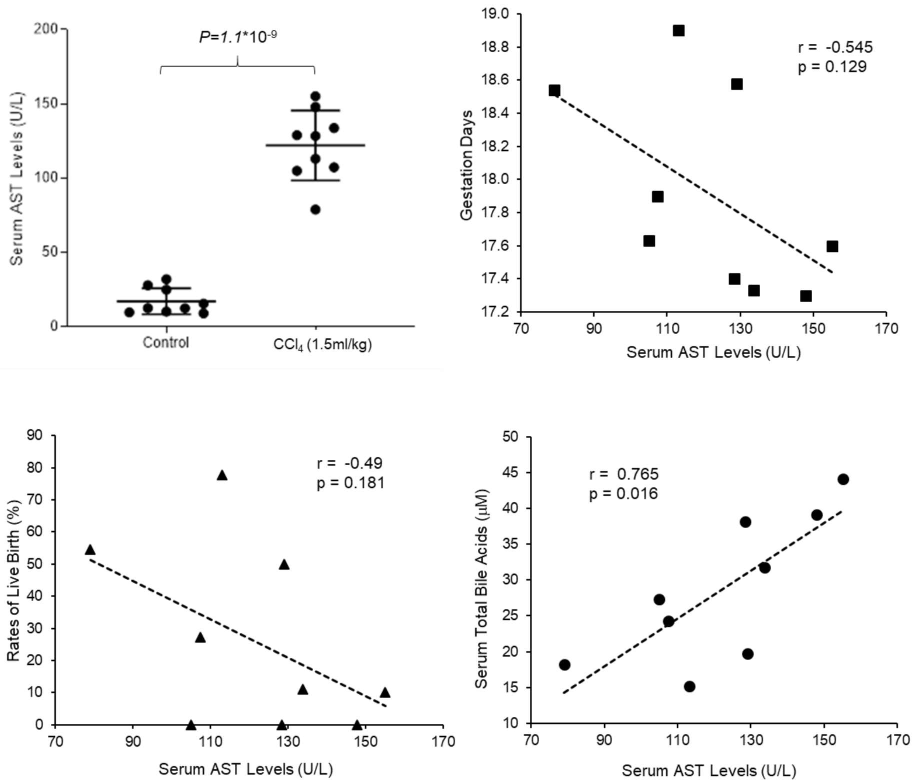

b

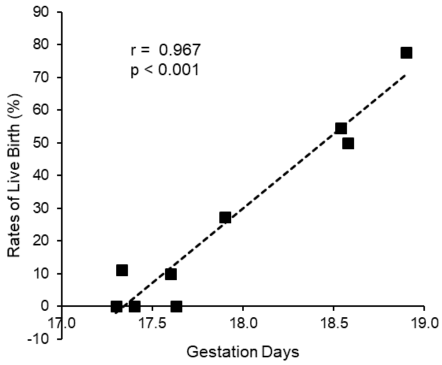

Supplementary Fig. 5. Elevated serum AST levels in CCl_4-inudced liver injuries and PTB. (**a**) serum AST levels in control (n=9) and CCl_4-treated mice (n=9), and correlation analyses between serum AST levels and gestation days, the rates of live birth and the sTBA levels. The data points for serum AST levels are presented as mean ± SD in the upper left panel. (**b**) a direct strong correlation between the gestation days and the rates of live birth was detected in the CCl_4-treated mice. The means and standard errors of the group values were indicated by the long and short lines, respectively. Student's t-test for pairwise comparison (two-sided) and Pearson correlation test were applied for statistical analysis. Source data are provided as a Source Data file.

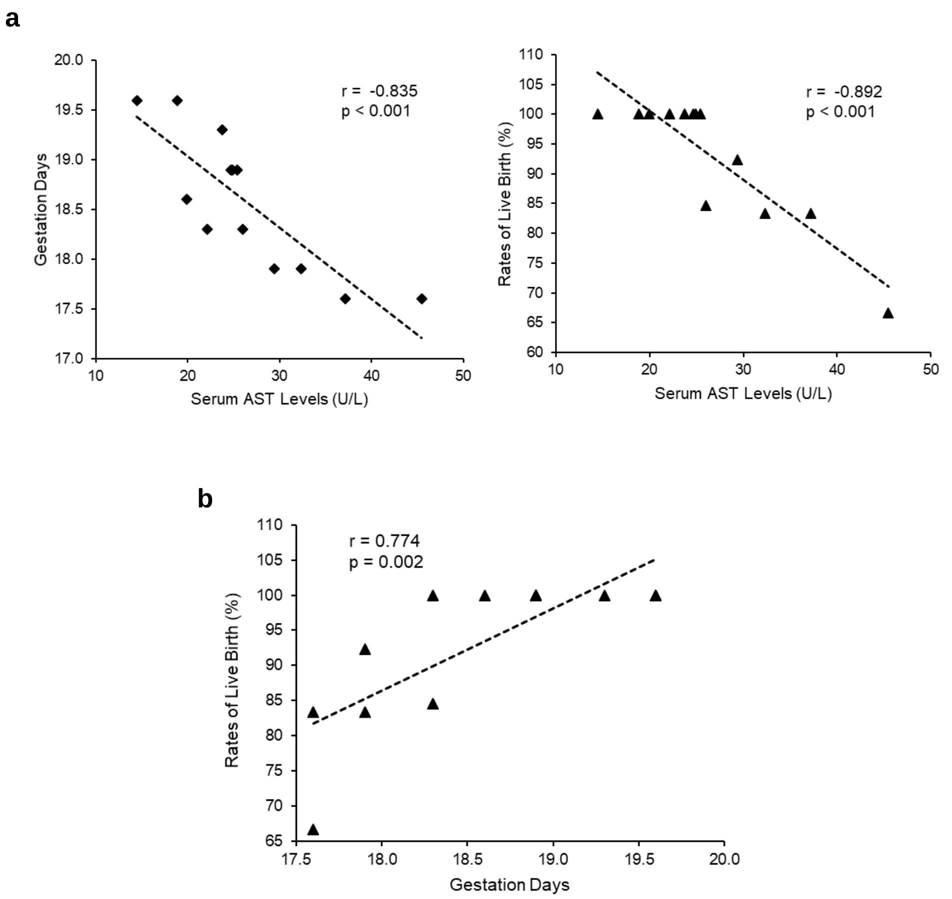

Supplementary Fig. 6. Serum AST levels correlated with gestation days and the rates of live birth in CA-treated mice. (**a**) a strong positive correlation between serum AST levels and the gestation days or the rates of live birth was detected in mice treated with CA-containing diet (n=13). (**b**) a positive correlation between the gestation days and the rates of live birth was detected in CA-treated mice. Pearson correlation analysis was applied to determine the correlation coefficient and associated p values. Source data are provided as a Source Data file.

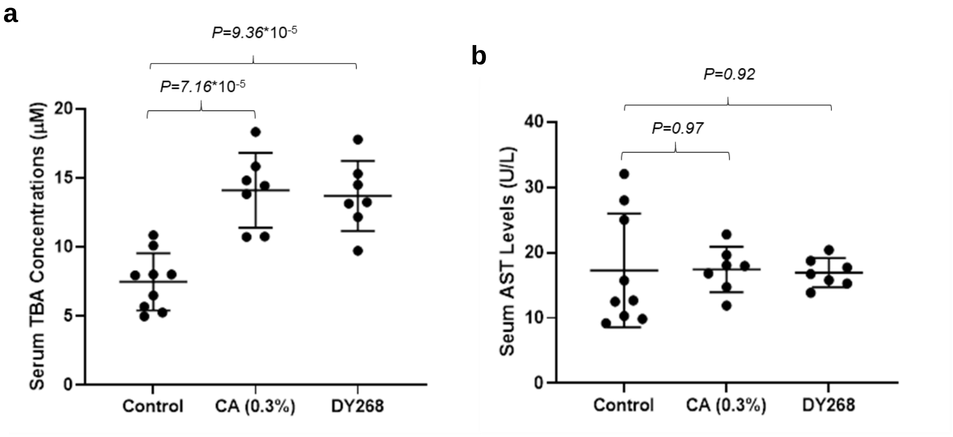

Supplementary Fig. 7. Elevated sTBA but AST levels in mice treated with 0.3% CA in diet or FXR antagonist DY268. (**a**) the sTBA levels were significantly elevated in mice treated with 0.3% CA in diet (n=7) or DY268 (20mg/kg) (n=7) when compared to the control group (n=9). The data points for serum TBA levels are presented as mean ± SD. (**b**) the AST levels in control (n=9) and mice treated with 0.3% CA (n=7) or DY268 (n=7). The AST levels were comparable among the three groups, indicating no liver injury by the treatment. The data points for serum AST levels are presented as mean ± SD. One-way ANOVA, followed by Tukey post-hoc test was applied for multiple group comparison. Source data are provided as a Source Data file.

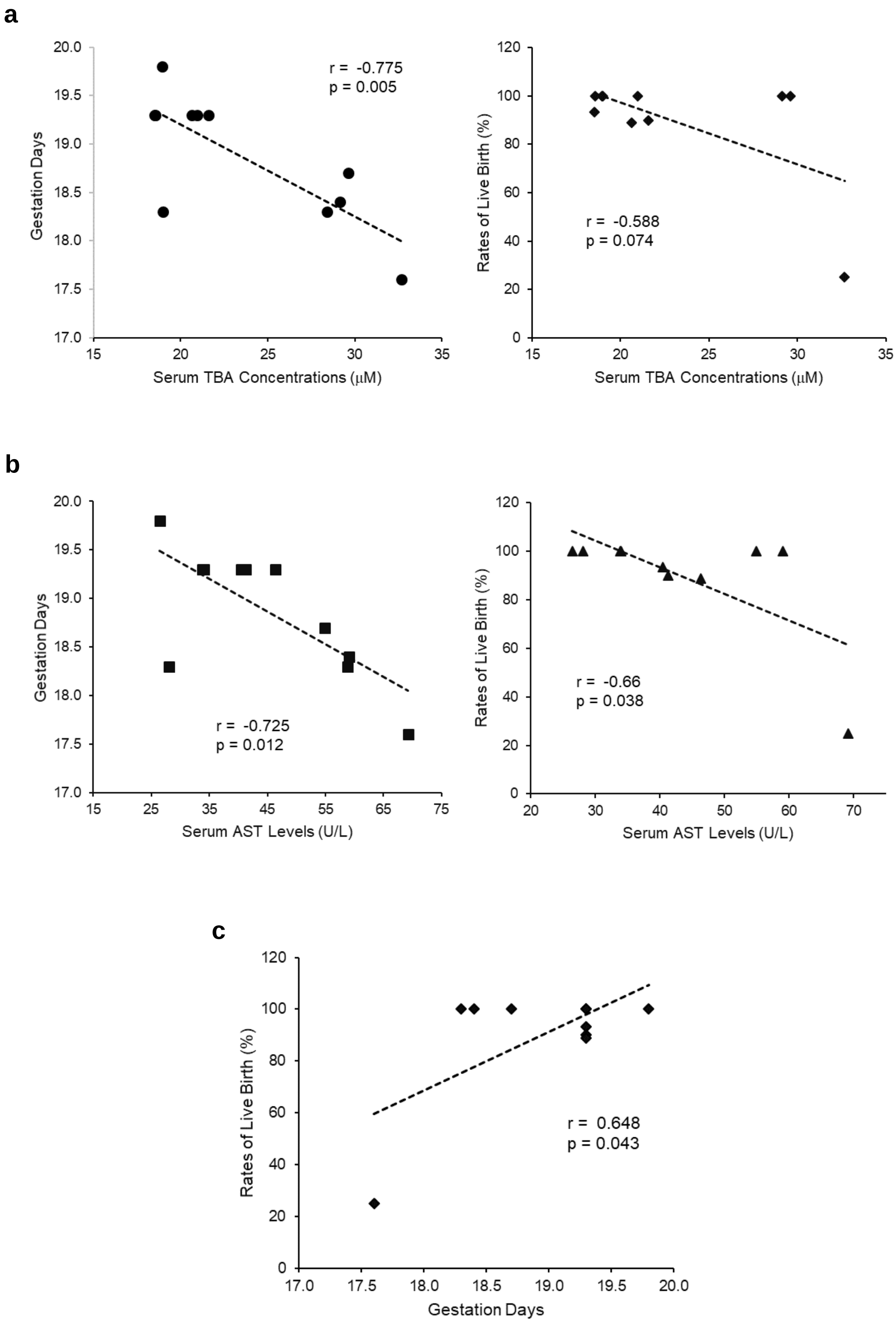

Supplementary Fig. 8. Correlation analyses among sTBA, AST, gestation days and rates of live birth in mice treated with a combination of CCl_4 and FXR agonist GW4064. (**a**) the sTBA levels positively correlated with the gestation days but not the rates of live birth in mice treated with CCl_4 and GW4064 (n=13). (**b**) serum AST levels directly correlated with the gestation days and the rates of live birth. (**c**) a positive correlation between the gestation days and the rates of live birth was detected. Pearson correlation analysis was applied to determine the correlation coefficient and associated p values. Source data are provided as a Source Data file.

Supplementary Table 1. Elevated sTBA levels were associated with increased risks for PTB including sPTB and iPTB.

Serum TBA (μM)	Total Subjects (n)	PTB Subjects (n)	Rates (%)	RR (95% CI)	p value	Serum AST (U/L)
**Total PTB**						
<10	34,227	2,605	7.61	1		15.5±9.4
10-39.9	2,362	217	9.19	1.20 (1.05-1.39)	0.0077	28.1±39.0
40-99.9	146	43	29.45	3.87 (2.86-5.23)	1.3×10^{-18}	83.3±96.9
≥100	20	10	50	6.57 (3.53-12.22)	2.8×10^{-9}	137.2±150.0
Total	36,755	2,875	7.82			20.4 ± 28.6
**sPTB**						
<10	34,227	1212	3.5	1		16.0 ± 11.9
10-39.9	2,362	71	3.0	0.85 (0.7-1.1)	0.18	38.4 ± 58.2
40-99.9	146	15	10.3	2.90 (1.7-4.8)	$4.1\text{x}10^{-5}$	72.9 ± 51.6
≥100	20	4	20.0	5.65 (2.1-15.1)	$5.5\text{x}10^{-4}$	117.3 ± 159.4
Total	36,755	1302	3.5			18.2 ± 22.2
**iPTB**						
<10	34,227	1393	4.1	1		17.8 ± 18.4
10-39.9	2,362	146	6.2	1.51 (1.3-1.8)	$1.5\text{x}10^{-6}$	46.2 ± 60.1
40-99.9	146	28	19.2	4.71 (3.2-6.9)	$4.6\text{x}10^{-16}$	92.1 ± 112.3
≥100	20	6	30.0	7.37 (3.3-16.4)	$1.0\text{x}10^{-6}$	111.7 ± 112.8
Total	36,755	1573	4.3			22.2 ± 28.6

sTBA, serum total bile acid; PTB, preterm birth; sPTB, spontaneous PTB; iPTB, iatrogenic PTB; AST, aspartate aminotransferase; RR, relative risk; CI, confidence interval. All the statistical analyses are two-sided.

Supplementary Table 2. Advanced maternal ages and increased pre-pregnant BMI were associated with increased risks for PTB and elevated sTBA and AST levels.

Subject Characteristics	Total Subjects (n)	PTB Subjects (n)	PTB Rates (%)	p Value	Serum Bile Acids (μM)	Serum AST (U/L)
**Maternal Age** (Year)						
18-24	9,000	730	8.1	1.3×10^{-4}	4.69 ± 5.51	16.46 ± 16.44
25-29	19,289	1,320	6.8		4.77 ± 6.46	16.57 ± 15.89
30-34	6,122	529	8.6	2.4×10^{-6}	4.71 ± 5.57	16.86 ± 16.51
35-39	2,025	232	11.5	2.9×10^{-15}	4.97 ± 7.29	17.64 ± 16.71
40-50	319	64	20.1	6.1×10^{-21}	5.60 ± 9.46	19.23 ± 23.22
**Pre-pregnant BMI**						
<18.5	2,973	243	8.2	0.03	4.67 ± 7.02	16.70 ± 17.45
18.5-24.9	29,004	2058	7.1		4.74 ± 5.98	16.49 ± 15.99
25-29.9	4,593	547	11.9	8.9×10^{-31}	4.89 ± 6.77	17.70 ± 16.96
≥30	185	27	14.6	7.9×10^{-5}	5.12 ± 7.86	19.62 ± 17.82

sTBA, serum total bile acid; PTB, preterm birth; AST, aspartate aminotransferase; BMI, body mass index. All the statistical analyses are two-sided.

Supplementary Table 3. ICP and NAFLD were associated with increased risks for PTB and elevated sTBA and AST levels.

Subject Characteristics	Subjects (n)	Rates (%)	Serum Bile Acids (μM)	p Value	Serum AST (U/L)	P Value
**All Subjects**						
FTB	33,880	92.18	4.68±5.31		16.36±14.70	
PTB	2,875	7.822	5.69±12.45	$4.2x10^{-18}$	20.35±28.56	$<10^{-31}$
sPTB	1,302	3.54	5.16±12.45	0.0027	18.18±22.18	$1.7x10^{-5}$
iPTB	1,573	4.28	6.13±13.56	$2.6x10^{-22}$	22.15±32.82	$<10^{-31}$
Total	36,755					
**NAFLD**						
FTB	1,337	86.04	6.53 ± 10.28		26.20 ± 34.51	
PTB	217	13.96	11.83 ± 22.01	$1.1x10^{-9}$	44.23 ± 56.97	$1.9x10^{-10}$
sPTB	85	5.47	11.57 ± 24.06	0.0001	39.72 ± 53.44	$7.8x10^{-4}$
iPTB	132	8.94	11.99 ± 20.68	$2.8x10^{-7}$	47.13 ± 59.14	$1.0x10^{-9}$
Total	1,554					
**ICP**						
FTB	388	76.83	28.33 ± 24.37		69.15 ± 84.22	
PTB	117	23.17	42.92 ± 42.44	$3.6x10^{-6}$	83.16 ± 91.38	0.12
sPTB	31	6.14	46.54 ± 44.38	0.0002	84.58 ± 90.34	0.33
iPTB	86	17.03	41.62 ± 41.92	0.0001	82.65 ± 92.27	0.19
Total	505					

sTBA, serum total bile acid; FTB, full-term birth; PTB, preterm birth; sPTB, spontaneous preterm birth; iPTB, iatrogenic preterm birth; AST, aspartate aminotransferase; ICP, intrahepatic cholestasis of pregnancy; NAFLD, non-alcohol fatty liver disease. All the statistical analyses are two-sided.
