# Supplementary material for: Dysregulation of bile acids increases the risk for preterm birth in pregnant women
Source: Nat Commun. 2020 Apr 30;11:2111. doi: 10.1038/s41467-020-15923-4 (PMC7193585; doi:10.1038/s41467-020-15923-4)
Supplement: Supplementary file 2 — Reporting Summary [file 41467_2020_15923_MOESM2_ESM.pdf]

nature research

Corresponding author(s): Ruitang Deng

Last updated by author(s): March 23, 2020

# Reporting Summary

Nature Research wishes to improve the reproducibility of the work that we publish. This form provides structure for consistency and transparency in reporting. For further information on Nature Research policies, see Authors & Referees and the Editorial Policy Checklist .

## Statistics

For all statistical analyses, confirm that the following items are present in the figure legend, table legend, main text, or Methods section.

| n/a | Confirmed | |
|---|---|---|
| ☐ | ☒ | The exact sample size (*n*) for each experimental group/condition, given as a discrete number and unit of measurement |
| ☐ | ☒ | A statement on whether measurements were taken from distinct samples or whether the same sample was measured repeatedly |
| ☐ | ☒ | The statistical test(s) used AND whether they are one- or two-sided<br>*Only common tests should be described solely by name; describe more complex techniques in the Methods section.* |
| ☒ | ☐ | A description of all covariates tested |
| ☒ | ☐ | A description of any assumptions or corrections, such as tests of normality and adjustment for multiple comparisons |
| ☐ | ☒ | A full description of the statistical parameters including central tendency (e.g. means) or other basic estimates (e.g. regression coefficient) AND variation (e.g. standard deviation) or associated estimates of uncertainty (e.g. confidence intervals) |
| ☐ | ☒ | For null hypothesis testing, the test statistic (e.g. *F*, *t*, *r*) with confidence intervals, effect sizes, degrees of freedom and *P* value noted<br>*Give P values as exact values whenever suitable.* |
| ☒ | ☐ | For Bayesian analysis, information on the choice of priors and Markov chain Monte Carlo settings |
| ☒ | ☐ | For hierarchical and complex designs, identification of the appropriate level for tests and full reporting of outcomes |
| ☒ | ☐ | Estimates of effect sizes (e.g. Cohen's *d*, Pearson's *r*), indicating how they were calculated |

*Our web collection on statistics for biologists contains articles on many of the points above.*

## Software and code

Policy information about availability of computer code

| | |
|---|---|
| Data collection | No specific codes were used to collect data. Human serum bile acids, AST, ALT, total bilirubin, GGT levels were measured and collected by the automatic biochemical analyzer (AU2700, Olympus). The mouse serum bile acids and AST levels were measured and collected by the GloMax® 96 Microplate Luminometer (Promega). |
| Data analysis | IBM SPSS Statistics 25 and SAS software were used for statistical analyses. Total serum bile acids, serum AST, ALT, total bilirubin, GGT and Real-time PCR data were analyzed using Prism version 8 (Graphpad software). |

For manuscripts utilizing custom algorithms or software that are central to the research but not yet described in published literature, software must be made available to editors/reviewers. We strongly encourage code deposition in a community repository (e.g. GitHub). See the Nature Research guidelines for submitting code & software for further information.

## Data

Policy information about availability of data

All manuscripts must include a data availability statement. This statement should provide the following information, where applicable:

- Accession codes, unique identifiers, or web links for publicly available datasets
- A list of figures that have associated raw data
- A description of any restrictions on data availability

All the processed data are available from the corresponding author upon request.

# Field-specific reporting

Please select the one below that is the best fit for your research. If you are not sure, read the appropriate sections before making your selection.

# Life sciences study design

All studies must disclose on these points even when the disclosure is negative.

| | |
|---|---|
| Sample size | A minimal of 5 mice per group were based on power calculation with assumption that there is a minimal of 20% difference between the groups. The heterogeneity of the sample sizes from 5 to 13 mice per group in the study reflects the variation in synchronized pregnancy rates during each experiment. |
| Data exclusions | No data were excluded for the analyses, but on rare occasion the newborn pups were eaten by the mother immediately after birth therefore the data on the rates of alive birth were not available (not included in the analyses). Also on rare occasion, limited amount of blood samples were collected due to technical issues from a mouse, the data on serum bile acids, AST concentrations or both were not available for the mouse (not included in the analyses). |
| Replication | The data derived from single dose studies were obtained from at least two independently repeated experiments. The data derived from the dose-response studies were obtained from single experiments considering that different doses can serve as a confirmation for each other. The mouse study with a combined treatment of 0.3% CA and DY268 in pregnant mice was performed once considering that the groups of mice treated with 0.3% CA or DY268 alone were included in the study. |
| Randomization | Randomization was applied for the studies with multiple groups. |
| Blinding | For the human study, data collection and analysis were blinded. For most of the animal studies, researchers were not blinded to the treatment, sample collection, data analysis as our studies utilized well-defined quantitative assays and did not involve any subjective measurements. For the histopathology study, the pathologist who evaluated the liver slides was blinded. |

# Reporting for specific materials, systems and methods

We require information from authors about some types of materials, experimental systems and methods used in many studies. Here, indicate whether each material, system or method listed is relevant to your study. If you are not sure if a list item applies to your research, read the appropriate section before selecting a response.

## Materials & experimental systems

| n/a | Involved in the study |
|---|---|
| [x] | [ ] Antibodies |
| [x] | [ ] Eukaryotic cell lines |
| [x] | [ ] Palaeontology |
| [ ] | [x] Animals and other organisms |
| [ ] | [x] Human research participants |
| [x] | [ ] Clinical data |

## Methods

| n/a | Involved in the study |
|---|---|
| [x] | [ ] ChIP-seq |
| [x] | [ ] Flow cytometry |
| [x] | [ ] MRI-based neuroimaging |

## Animals and other organisms

Policy information about studies involving animals; ARRIVE guidelines recommended for reporting animal research

| | |
|---|---|
| Laboratory animals | All animals used in the study are CD-1 mice during reproductive age of 3 to 8 months. Pregnant female mice were used in the studies while male mice were used only for the purpose of mating. |
| Wild animals | No wild animals were used in the study. |
| Field-collected samples | No field-collected samples were used in the study. |
| Ethics oversight | The Institutional Animal Care and Use Committee (IACUC) of the University of Rhode Island. |

Note that full information on the approval of the study protocol must also be provided in the manuscript.

## Human research participants

Policy information about studies involving human research participants

| | |
|---|---|
| Population characteristics | A total of 36,755 pregnant women were included in the study aged 18 to 50 years. |
| Recruitment | Written informed consents were obtained from all participants. |
| Ethics oversight | The Institutional Review Board (IRB) of Nantong Maternal and Child Health Hospital. |

Note that full information on the approval of the study protocol must also be provided in the manuscript.
